# Insights into the Discovery of Novel Neuroprotective Agents: A Comparative Study between Sulfanylcinnamic Acid Derivatives and Related Phenolic Analogues

**DOI:** 10.3390/molecules24234405

**Published:** 2019-12-02

**Authors:** Daniel Chavarria, Carlos Fernandes, Brandon Aguiar, Tiago Silva, Jorge Garrido, Fernando Remião, Paulo J. Oliveira, Eugenio Uriarte, Fernanda Borges

**Affiliations:** 1 CIQUP/Department of Chemistry and Biochemistry, Faculty of Sciences, University of Porto, 4169-007 Porto, Portugal; danielf_chavarria@live.com.pt (D.C.); cevinhas@gmail.com (C.F.); brandonlageaguiar@gmail.com (B.A.); ntb.silva@gmail.com (T.S.); 2 CNC–Center for Neuroscience and Cell Biology, University of Coimbra, UC Biotech, Biocant Park, 3060-197 Cantanhede, Portugal; pauloliv@cnc.uc.pt; 3 Department of Chemical Engineering, School of Engineering (ISEP), Polytechnic of Porto, 4200-072 Porto, Portugal; jjg@isep.ipp.pt; 4 UCIBIO-REQUIMTE, Laboratory of Toxicology, Department of Biological Sciences, Faculty of Pharmacy, University of Porto, 4050-313 Porto, Portugal; remiao@ff.up.pt; 5 Departamento de Química Orgánica, Facultad de Farmacia, 15782 Santiago de Compostela, Spain; eugenio.uriarte@usc.es; 6 Instituto de Ciencias Químicas Aplicadas, Universidad Autónoma de Chile, Santiago 8900000, Chile

**Keywords:** cinnamic acid, 4-thioferulic acid derivatives, ferulic acid derivatives, antioxidant activity, cytotoxicity, neuroprotection

## Abstract

Exogenous antioxidants may be beneficial therapeutic tools to tackle the oxidative damage in neurodegenerative diseases by regulation of the redox state that is critical for cell viability and organ function. Inspired by natural plant polyphenols, a series of cinnamic acid-based thiophenolic and phenolic compounds were synthesized and their antioxidant and neuroprotective properties were studied. In general, our results showed that the replacement of the hydroxyl group (OH) by a sulfhydryl group (SH) increased the radical scavenging activity and enhanced the reaction rate with 1,1-diphenyl-2-picrylhydrazyl radical (DPPH^^•^^) and galvinoxyl radical (GO^^•^^). These results correlated well with the lower oxidation potential (*E*_p_) values of thiophenols. However, a lower peroxyl radical (ROO^^•^^) scavenging activity was observed for thiophenols in oxygen radical absorbance capacity (ORAC-FL) assay. Furthermore, the introduction of 5-methoxy and 5-phenyl groups in the aromatic ring of 4-thioferulic acid (TFA) **2** and ferulic acid (FA) **1** did not significantly improve their antioxidant activity, despite the slight decrease of *E*_p_ observed for compounds **5**, **6,** and **9**. Concerning cinnamic acid amides, the antioxidant profile was similar to the parent compounds. None of the compounds under study presented significant cytotoxic effects in human differentiated neuroblastoma cells. Thiophenolic amide **3** stands out as the most promising thiophenol-based antioxidant, showing cellular neuroprotective effects against oxidative stress inducers (hydrogen peroxide and iron).

## 1. Introduction

Oxidative stress is currently defined as the deregulation of the balance between the oxidants and antioxidants in favor of the former, with consequential disruption of the redox signaling and/or molecular damage [1]. The harmful effects on human cells associated with the oxidative damage inflicted by reactive species (RS) may ultimately lead to programmed cell death [2]. Several lines of evidence implicate oxidative stress on the neurodegeneration process observed in patients with Alzheimer’s disease (AD) and Parkinson’s disease (PD) [3]. Actually, markers of lipid peroxidation and protein oxidation were detected in individuals with AD and PD [4,5,6,7,8]. Both disorders were also associated with a decline of the pool of antioxidants, and a decrease of the activity of antioxidant enzymes [9,10,11,12].

Given the role of oxidative stress on the pathogenesis of neurodegenerative diseases, the use of exogenous antioxidants to assist the antioxidant defense system in upstream (prevention of RS generation) or downstream (radical scavenging) antioxidant pathways may help to maintain neuronal integrity and delay the neurodegenerative processes [13,14]. 

**Figure 1:**
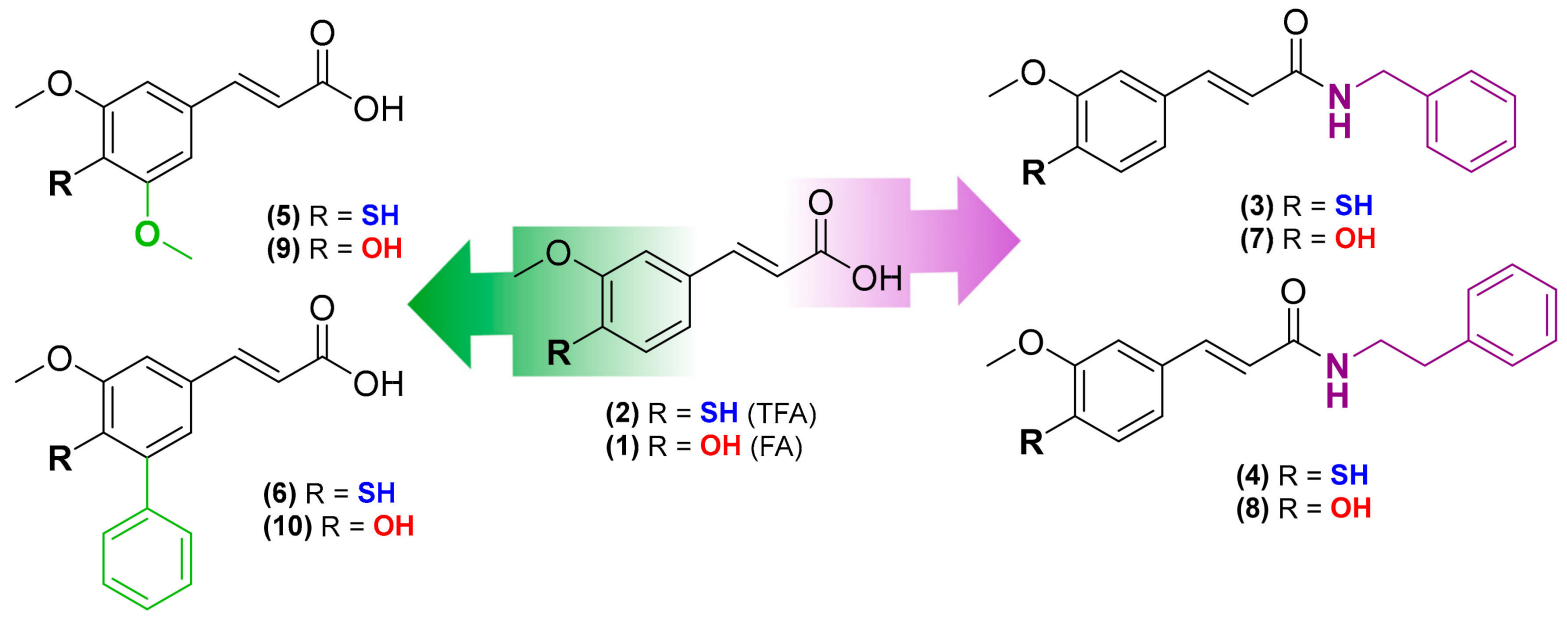
Rational design of new chemical entities based on FA (compound **1**) and TFA (compound **2**) scaffolds.

Ferulic acid (3-(4-hydroxy-3-methoxyphenyl)-2-propenoic acid, FA, compound **1**, Figure 1) is a natural hydroxycinnamic acid (HCA) able to act directly by scavenging harmful oxidants, or indirectly by downregulation of pathways involved in cell-death and upregulation of the expression of antioxidant enzymes [15]. Neuroprotective properties in both in vitro and in vivo models, associated with antioxidant and anti-inflammatory activities, were ascribed to FA [16,17,18]. However, the therapeutic use of FA is hampered by its poor bioavailability, as a consequence of its low absorption and rapid metabolism and excretion [19,20]. 

Ferulic acid has been used as a scaffold for the design and development of novel antioxidants with potential therapeutic interest [21,22,23]. Within this framework, 4-thioferulic acid (TFA, compound **2**, Figure 1), a new antioxidant obtained by the isosteric replacement of the hydroxyl group (OH) by a sulfhydryl group (SH) in FA, was reported by our group [24]. 

To gain insight on the advantages of thiophenol systems, herein we report the synthesis and the biological screening of novel TFA derivatives (compounds **3**–**6**, Figure 1). The corresponding hydroxycinnamic acid derivatives (compounds **7**–**10**, Figure 1) were synthesized to directly compare the effect of the OH to SH replacement. Following the studies of the antioxidant activity, we assessed the cytotoxicity of the new derivatives in differentiated SH-SY5Y cells and their protective properties against oxidative stress insults (H_2_O_2_, iron) in the same cellular model. 

## 2. Results and Discussion

### 2.1. Chemistry

**Scheme 1:**
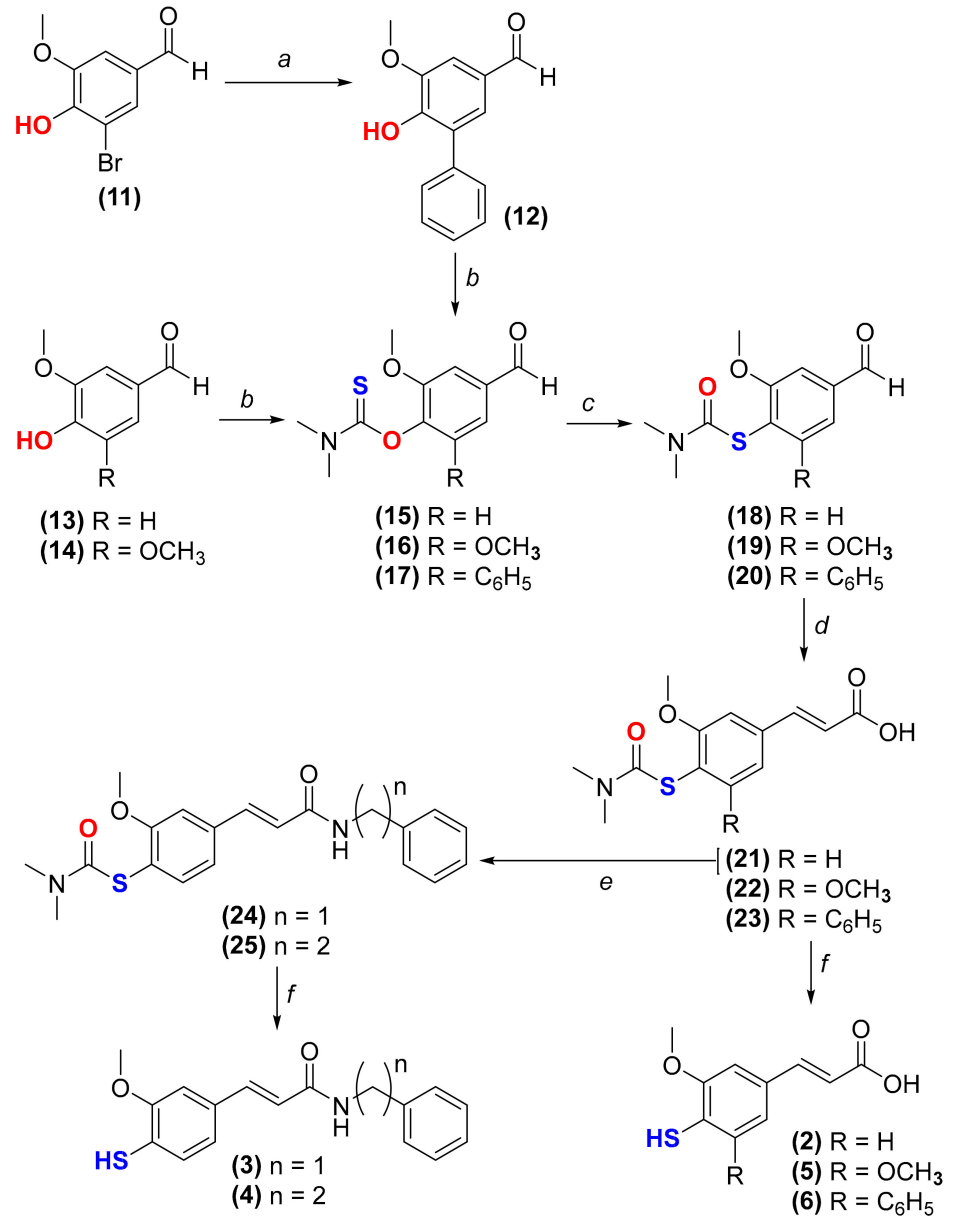
Synthetic strategy followed for the synthesis of compounds **2**–**6**. (**a**) phenylboronic acid, K_2_CO_3_, Pd(OAc)_2_, TBAB, DMF, 150 °C (microwave (MW) irradiation), 15 min; (**b**) DMTCl, DABCO, DMF, 70 °C, 3 h; (**c**) diphenyl ether, argon, 240 °C (MW irradiation), 20 min; (**d**) malonic acid, anhydrous pyridine, piperidine, 70 °C, 4–5 h; (**e**) 1. DMF, DIPEA, PyBOP, CH_2_Cl_2_, 0 °C, 30 min; 2. benzylamine or 2-phenylethylamine, rt, overnight; (f) MeOH, aqueous solution of NaOH 2 M, reflux, 1.5–16 h.

The synthesis of TFA (compound **2**) [24] and derivatives thereof (compounds **3**–**6**) was performed following the strategy depicted in Scheme 1. First, the reaction between 5-bromovanillin (compound **11**) and phenylboronic acid in the presence of Pd(OAc)_2_ and K_2_CO_3_ under microwave (MW) irradiation yielded 5-phenylvanillin (compound **12**) (Scheme 1, step *a*). Then, the thiocarbamoylation of 4-hydroxybenzaldehyde derivatives **12**–**14** with dimethylthiocarbamoyl chloride (DMTCl) under alkaline conditions afforded compounds **15**–**17** (Scheme 1, step *b*). A MW-assisted Newman-Kwart rearrangement under inert atmosphere of compounds **15**–**17** yielded compounds **18**–**20** (Scheme 1, step *c*). Cinnamic acid derivatives **21**–**23** were obtained via Knoevenagel-Doebner condensation between aldehydes **18**–**20** and malonic acid (Scheme 1, step *d*). The PyBOP-mediated amidation of compound **21** with benzylamine or phenethylamine led to the obtainment of the amides **24** and **25**, respectively (Scheme 1, step *e*). Finally, the alkaline hydrolysis of compounds **21**–**25** afforded thiophenol derivatives **2**–**6** (Scheme 1, step *f*).

The corresponding hydroxycinnamic acid derivatives (compounds **7**–**10**, Figure 1) were synthesized according to procedures previously reported [21,25].

### 2.2. Evaluation of Antioxidant Activity

Following the synthesis of TFA and FA derivatives (compounds **1**–**10**), we evaluated their radical scavenging activity against three synthetic radicals: 1,1-diphenyl-2-picrylhydrazyl radical (DPPH^•^), 2,2′-azinobis-(3-ethylbenzothiazoline-6-sulfonic acid) radical cation (ABTS^^•^+^) and galvinoxyl (GO^•^). In these assays, after the generation of the synthetic colored radical, the radical scavenging activity is evaluated by spectrophotometry [26]. Trolox was used as a reference antioxidant. The results are presented as mean IC_50_ ± SD (*n* = 3) values (Table 1). 

In general, thiophenols **2**–**6** showed higher scavenging of DPPH^•^ and GO^•^ radicals than phenols **1, 7**–**10**, while the scavenging of ABTS^^•^+^ radical was similar. The introduction of an additional 5-methoxy group (compound **5**) was proven to be a structural modification with limited benefits, since it only caused slight changes in the antioxidant activity of TFA (compound **2**) (a modest improvement in the antioxidant activity was only observed in DPPH^•^assay). Conversely, the presence of an additional aromatic ring (compound **6**) decreased the antioxidant activity of TFA. Although an additional aromatic ring can increase the electron delocalization and stabilize the generated thiophenoxyl radical by resonance, it may reduce the accessibility of the compound to the center of the radicals and thereby decrease the radical scavenging activity [27]. The same tendency was observed with the phenolic analogues.

Considering that both 5-methoxy and 5-phenyl groups did not significantly increase the radical scavenging activity of TFA, we then evaluated the effect of the introduction of substituents on the carboxylic function of TFA. As shown in Table 1, benzyl and phenethyl amides of TFA (compounds **3** and **4,** respectively) displayed similar IC_50_ values to the free carboxylic acid. The same tendency was observed for the phenolic analogues (compounds **7** and **8**). Therefore, the carboxylic acid amidation with lipophilic substituents maintained the antioxidant activity of TFA.

The kinetic data obtained in DPPH^•^, ABTS^^•^+^, and GO^•^ assays can provide information concerning the reactivity of the compounds under study (Figures S1–S3). As previously reported for TFA [24], thiophenols **3**–**6** reacted almost instantaneously with the radicals, leading to fast absorbance decays and reaching the steady state within the first minutes of reaction (Figures S1A–E, S2A–E and S3A–E). These results pointed out that the reaction rate was increased by the OH to SH replacement. Compared to TFA, the reactions of compounds **5** and **6** with the radicals occurred slower, requiring more time to reach the steady state (Figures S1D,E, Figures S2D,E and Figures S3D,E). Therefore, the presence of 5-methoxy and 5-phenyl groups in thiophenols delayed the reaction progress, possibly due to increased steric hindrance. On the other hand, compounds **3** and **4** displayed the same kinetic profile of TFA (Figures S1B,C, Figures S2B,C and Figures S3B,C), indicating that the reactivity of TFA was preserved with the functionalization of the carboxylic acid with lipophilic groups. Like ferulic acid **1** [24], phenols **7**–**10** displayed hyperbolic kinetic curves indicating moderate reactivity towards DPPH^•^, ABTS^^•^+^, and GO^•^ (Figures S1F–J, S2F–J and S3F–J).

We also studied the antioxidant activity toward peroxyl radicals (ROO^•^) using the oxygen radical absorbance capacity (ORAC-FL) assay. In this assay, ROO^•^ radicals are scavenged by hydrogen atom transfer (HAT) or by radical addition processes [27]. Phenols (compounds **1**, **7**–**10**) showed ORAC-FL indexes ranging between 2.7–4.0 (Table 1) and displayed higher ORAC-FL indexes than thiophenols (compounds **2**–**6**). Thiophenols presented ORAC-FL indexes below 1 (Table 1). Therefore, contrary to what was observed in DPPH^•^, ABTS^^•^+^ and GO^•^assays, these results show that phenols are better ROO^•^ radical scavengers than thiophenols. The dissimilarities between the two sets of compounds may be related to the differences on the acidity of the phenol and the thiophenol moieties. Since the thiophenol group of TFA **1** is significantly more acidic than the phenol group of FA (p*K*_a thiophenol_ = 5.6; p*K*_a phenol_ = 9.1) [24], there is a high amount of thiophenolate anions at pH 7.3 while the phenol group remains undissociated. Therefore, in opposition to phenols **1** and **7**–**10**, the deprotonated thiophenols **2**–**6** were poor scavengers of ROO^•^ radicals.

### 2.3. Electrochemical Studies

Voltammetric measurements are particularly useful to estimate the redox properties of bioactive systems, thus enabling a prompt screening of their antioxidant capacity [28]. In this context, to obtain complementary information concerning the antioxidant activity of the compounds under study, differential pulse voltammetry (DPV) experiments were performed to study the electrochemical properties of compounds **2**–**6** and **7**–**10**. Experiments were carried out at a physiological pH of 7.4 using a glassy carbon-working electrode. The oxidation potential (*E_p_*) values obtained are depicted in Table 1.

All compounds showed well-defined anodic waves at physiological pH, which may be attributed to the oxidation of the thiophenol or the phenol groups. Thiols and phenols can undergo one-electron oxidation processes with the formation of thiyl and phenoxyl radicals, respectively [29,30]. The *E_p_* values of thiol-containing compounds **2**–**6** ranged between +186 mV and +271 mV, while phenols **1** and **7**–**10** displayed higher *E*_p_ values, ranging between +291 mV and +351 mV (Table 1**)**. The results obtained showed that the replacement of the OH by an SH group shifted the peak potentials toward less positive values, suggesting that thiophenols are more prone to oxidation than phenols.

The introduction of 5-methoxy (compounds **5** and **9**) or 5-phenyl (compounds **6** and **10**) groups did not significantly change the *E*_p_ values when compared to FA and TFA, although a slight decrease was observed for compounds **5**, **6,** and **9**. The lower potential values may result from the increased stabilization of the thiophenoxyl/phenoxyl radicals, which is associated with the electron-donating properties of the methoxy group [31] (compounds **5** and **9**) or the increased electron delocalization provided by the additional aromatic ring (compound **6**).

The voltammetric behavior of the cinnamic acid amides **3**, **4**, **7**, and **8** was similar to that observed for the parent compounds (TFA and FA). The introduction of lipophilic groups at the side chain did not significantly affect the redox properties of phenol and thiophenol groups and, therefore, no major changes in the redox potentials were observed.

### 2.4. Cellular Studies

#### 2.4.1. Evaluation of Cytotoxicity Profile

**Figure 2:**
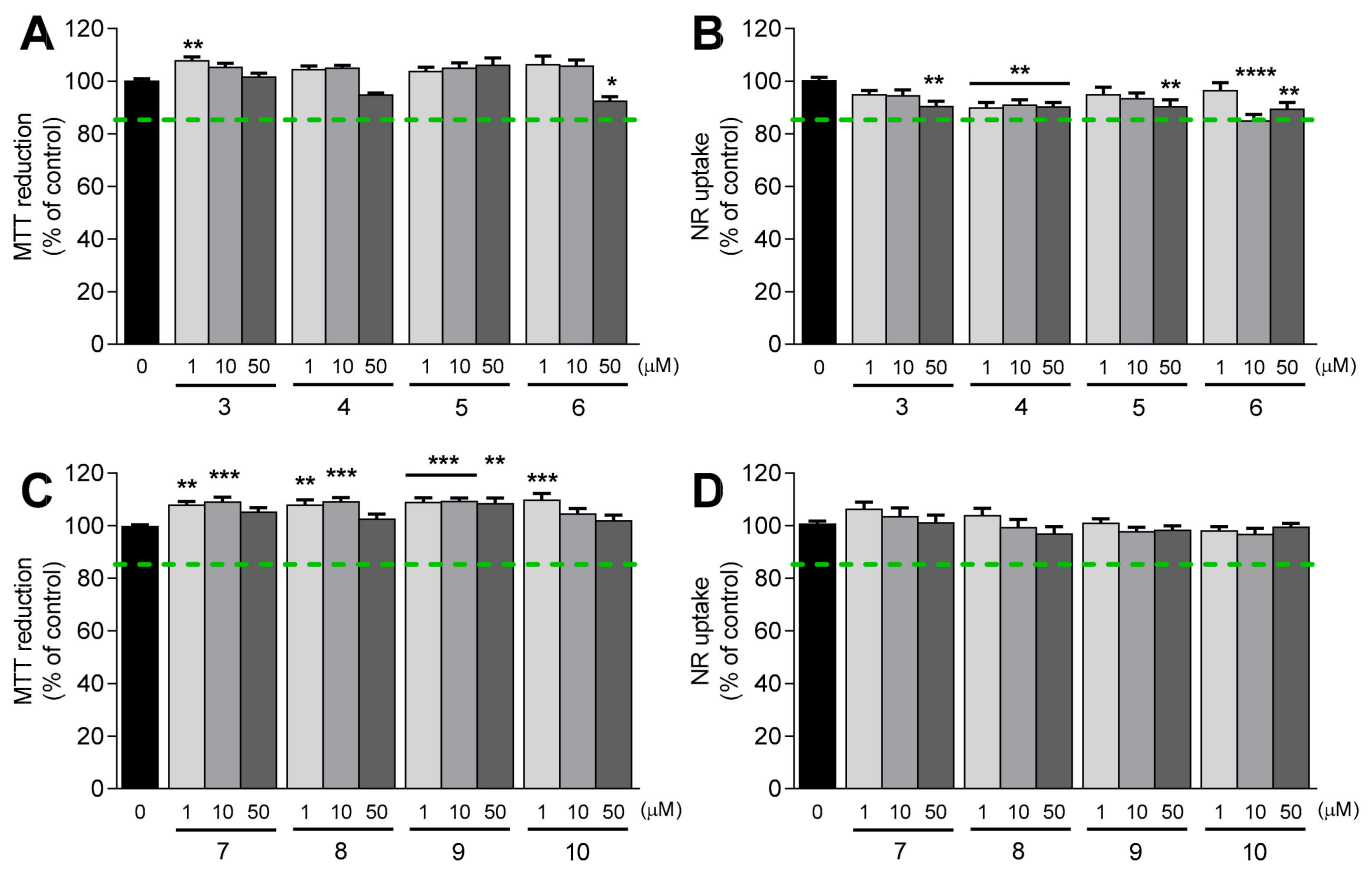
Cellular viability of differentiated SH-SY5Y cells after a 24 h treatment with thiophenols **3**–**6** and phenols **7**–**10** at three different concentrations (1, 10 and 50 µM). Cellular viability was evaluated using two methods: MTT reduction (**A**, **C**) and NR uptake (**B**, **D**) assays. Results are expressed as the mean % of untreated controls ± SEM (*n* = 3). Statistical comparisons were performed using the parametric method of one-way ANOVA, followed by the Dunnett’s multiple comparisons test. In all cases, *p* values lower than 0.05 were considered significant (* *p* < 0.05, ** *p* < 0.01, *** *p* < 0.001, **** *p* < 0.0001 vs untreated cells).

The cytotoxicity profile of thiophenols **3**–**6** and phenols **7**–**10** was assessed in human neuroblastoma SH-SY5Y cells differentiated into a dopaminergic phenotype [32]. After 24 h of exposure with the compounds at three different concentrations (1, 10 and 50 µM), the cell viability was evaluated using two methods: the 3-(4,5-dimethylthiazol-2-yl)-2,5-diphenyltetrazolium (MTT) reduction assay, a method that relies on the reduction of MTT into the respective formazan by cells with active metabolism [33], and the neutral red (NR) uptake assay, which is based on the lysosomal uptake of the dye NR [34]. The results, expressed as mean MTT reduction (% of control) SEM and NR uptake (% of control) ± SEM (*n* = 3), are depicted in Figure 2.

Thiophenols **3**–**6** (Figure 2A,B) and phenols **7**–**10** (Figure 2C,D) did not cause marked decreases in MTT reduction and in NR uptake at concentrations up to 50 µM (MTT reduction and NR uptake were >85%), which is in accordance with the previous data obtained for FA **1** and TFA **2** [24]. Based on these results, we can conclude that the cytotoxicity profile within the tested concentration range was upheld with the OH to SH replacement, the introduction of 5-methoxy and 5-phenyl substituents, and the functionalization of the carboxylic acid group (benzyl or phenethyl amides).

#### 2.4.2. Protection against H_2_O_2_- and Ferric Iron-Induced Oxidative Damage

**Figure 3:**
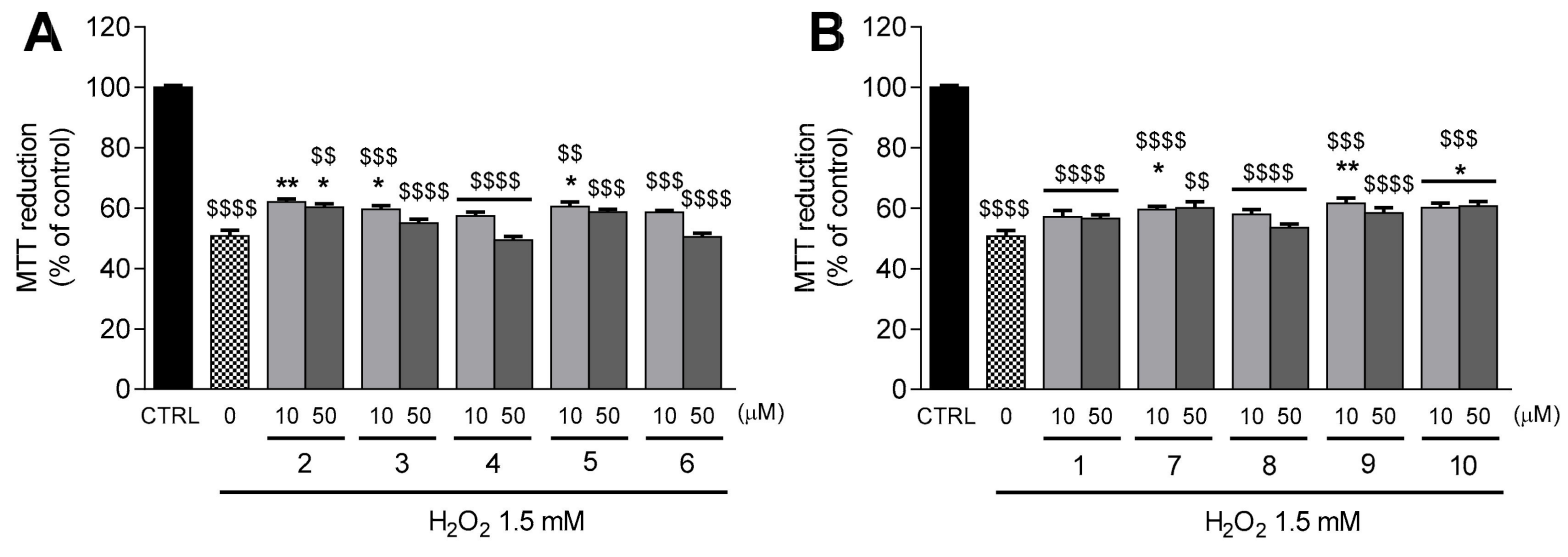
Protection against H_2_O_2_-induced damage in differentiated SH-SY5Y cells by thiophenols **2**–**6** (**A**) and phenols **1**, **7**–**10** (**B**). After a pre-treatment with the compounds under study (10 and 50 µM) for 24 h, cells were exposed H_2_O_2_ 1.5 mM for additional 30 min. Cellular viability was evaluated by the MTT reduction assay. Results are expressed as the mean % of untreated controls ± SEM (*n* = 3). Statistical comparisons were estimated using the nonparametric method of Kruskal–Wallis (one-way ANOVA on ranks), followed by Dunn’s *post hoc* test (* *p* < 0.05, ** *p* < 0.01, compared with cells treated only with H_2_O_2_; ^$$^ *p* < 0.01, ^$$$^ *p* < 0.001; ^$$$$^ *p* < 0.0001 compared with CTRL). Positive control with caffeic acid (50 µM) was performed under the same conditions described above. The result is depicted in Supplementary Information (Figure S4A).

**Figure 4:**
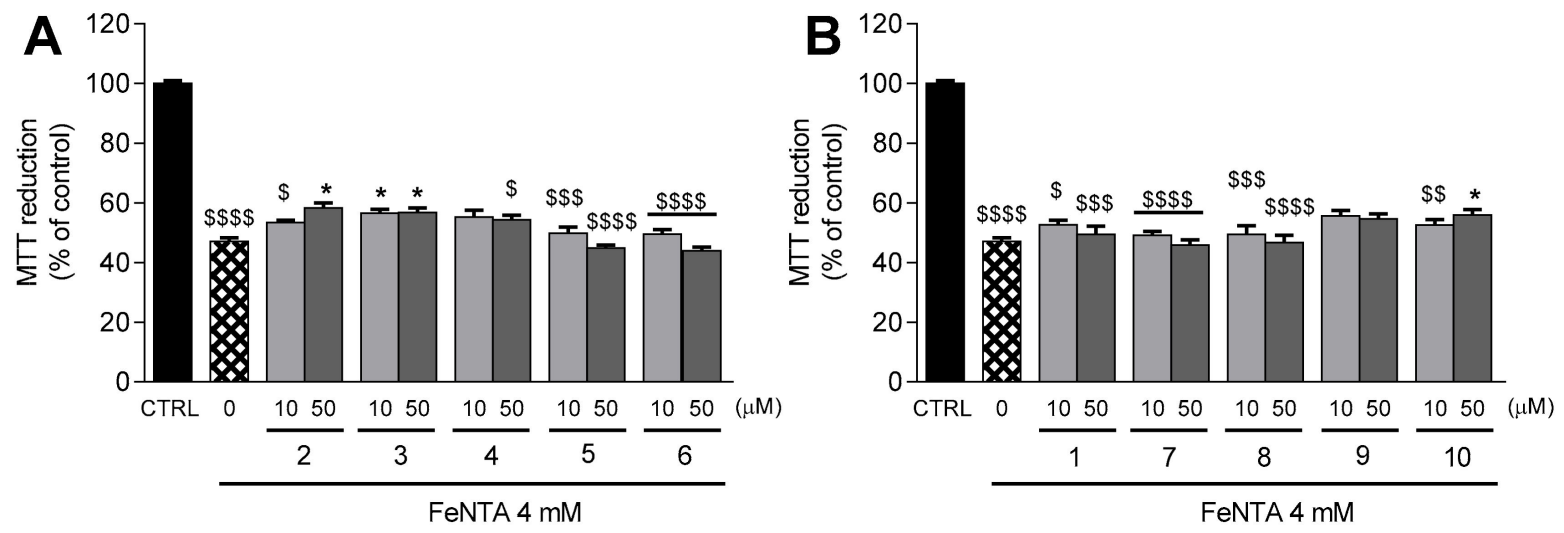
Protection against FeNTA-induced damage in differentiated SH-SY5Y cells by thiophenols **2**–**6** (**A**) and phenols **1**, **7**–**10** (**B**). After a pre-treatment with the compounds under study (10 and 50 µM) for 24 h, cells were exposed FeNTA 4 mM for additional 24 h. Cellular viability was evaluated by the MTT reduction assay. Results are expressed as the mean % of untreated controls ± SEM (*n* = 3). Statistical comparisons were estimated using the nonparametric method of Kruskal–Wallis (one-way ANOVA on ranks), followed by Dunn’s *post hoc* test (* *p* < 0.05 compared with cells treated only with FeNTA; ^$^ *p* < 0.05, ^$$^ *p* < 0.01, ^$$$^ *p* < 0.001; ^$$$$^ *p* < 0.0001 compared with CTRL). Positive control with caffeic acid (50 µM) was performed under the same conditions described above. The result is depicted in Supplementary Information (Figure S4B).

The protective properties of compounds **1**–**10** against oxidative stress in the same cellular model were also examined. Hydrogen peroxide (H_2_O_2_) and ferric nitrilotriacetate (FeNTA) were used as inducers of oxidative stress [32]. After the pre-treatment with the test compounds at 10 and 50 µM for 24 h, differentiated SH-SY5Y cells were exposed to H_2_O_2_ (1.5 mM) or FeNTA (4 mM) for 30 min and 24 h, respectively. The cellular viability was assessed using the MTT reduction assay. The results obtained are illustrated in Figure 3 and Figure 4. 

The incubation of differentiated SH-SY5Y cells with H_2_O_2_ (1.5 mM) decreased the MTT reduction to 50.7 ± 1.9% (*p* < 0.0001) in comparison to control cells (Figure 3A,B). The damage inflicted by H_2_O_2_ was attenuated by thiophenols and phenols containing benzyl amide (compounds **3** and **7**, *p* < 0.05) and 5-methoxy groups (compound **5**, *p* < 0.05, and compound **9**, *p* < 0.01) at 10 µM (Figure 3A,B). A mild protection against H_2_O_2_-induced cell death was also observed with pre-treatment of neuroblastoma cells with TFA (10 µM, *p* < 0.01; 50 µM, *p* < 0.05, Figure 3A) and compound **10** (*p* < 0.05, Figure 3A) at both tested concentrations. The data obtained for TFA **2** and FA **1** at 50 µM were previously reported by Chavarria et al. [24].

Transition metals like iron accumulate with age in specific brain areas, such as the s*ubstantia nigra* and the hippocampus [35]. FeNTA was used to mimic iron overload conditions in SH-SY5Y cells. Treatment with FeNTA 4 mM decreased the MTT reduction to 47.2 ± 1.2% (*p* < 0.0001) in comparison to control cells (Figure 4A,B). Remarkably, TFA derivative **3** significantly protected neuroblastoma cells from the deleterious effects of FeNTA at both tested concentrations (*p* < 0.05, Figure 4A). The FeNTA-induced decrease in MTT reduction was also attenuated in neuroblastoma cells pre-treated with TFA **2** (*p* < 0.05, Figure 4A) and compound **10** (*p* < 0.05, Figure 4B) at 50 µM. 

Taking these data together, we concluded that TFA **2**, TFA benzyl amide **3,** and FA derivative **10** significantly protected neuroblastoma cells against oxidative stress, both by the direct action of H_2_O_2_ and by high concentrations of iron. In addition, 5-methoxy derivatives (compounds **5** and **9**) and FA benzyl amide (compound **7**) were able to prevent the damage induced by H_2_O_2_, but were ineffective against the harmful effects caused by high concentrations of iron.

### 2.5. Estimation of Drug-Like Properties

In early drug discovery, the balancing of key physicochemical properties within optimal ranges may improve the compounds’ quality and thereby increase the likelihood of therapeutic success [36]. In this context, we calculated several physicochemical parameters of TFA (compound **2**) and compounds **3** and **10** to predict their “drug-likeness”. These compounds were considered the most promising cinnamic acid derivatives because they displayed significant neuroprotective activity against two oxidative stressors in neuroblastoma cells. The predicted physicochemical properties included topological polar surface area (TPSA in Å), number of hydrogen acceptors (HBA), number of hydrogen donors (HBD), number of rotatable bonds (RB), and logarithm of the ratio of the concentration of a drug in the brain and in the blood (logBB). The results obtained are presented in Table 2. 

The values obtained for compound **3** are within the optimal ranges of drugs able to act in the central nervous system (CNS). On the other hand, TFA and compound **10** presented cLogP and LogBB values below or close to the recommended limits. Therefore, TFA **2** and compound **10** are less likely to cross the blood-brain barrier by passive diffusion and thereby attain the CNS. The presence of a free carboxylic acid may restrict the access to the brain, as previously observed for similar systems [21].

Overall, these results suggest that TFA benzyl amide (compound **3**) presents the most favorable drug-like properties among the selected candidates. 

## 3. Materials and Methods 

### 3.1. Chemistry

#### 3.1.1. Synthesis

##### I. Synthesis of 6-Hydroxy-5-methoxy-[1,1′-biphenyl]-3-carbaldehyde (**12**)

Synthesis and structural characterization are described in literature [21].

##### II. Thiocarbamoylation of Phenols

In a bottom flask with the phenol (1 mmol) dissolved in DMF, DABCO (4 mmol), and DMTCl (2 mmol) were added. The mixture was heated at 70 °C for 3 h. Upon completion, the mixture was poured onto water (15 mL) cooled on an ice bath and acidified with HCl 6 M. The product was collected by filtration under reduced pressure and washed with water. The general procedure was adapted from Nowakowska et al. [39].

*O*-(4-Formyl-2-methoxyphenyl) dimethylcarbamothioate (**15**). Synthesis and structural analysis are described in literature [24,39].

*O-(4-Formyl-2,6-dimethoxyphenyl) dimethylcarbamothioate* (**16**). The reaction mixture was poured onto water (15 mL) cooled on an ice bath and acidified with HCl 6 M. The product was collected by filtration under reduced pressure and washed with water. η = 93%. ^1^H NMR (CDCl_3_-*d_1_*)*:* δ (ppm) = 3.40 (*s*, 3H, NCH_3_), 3.49 (*s*, 3H, NCH_3_), 3.93 (*s*, 6H, 2 × OCH_3_), 7.19 (*s*, 2H, H2, H6), 9.94 (*s*, 1H, CHO). ^13^C NMR (CDCl_3_-*d_1_*): δ (ppm) = 38.9 (NCH_3_), 43.5 (NCH_3_), 56.5 (2 × OCH_3_), 106.3 (C2, C6), 134.2 (C1), 136.9 (C4), 153.4 (C3, C5), 186.7 (CSO), 191.1 (CHO). EI/MS *m*/*z* (%): 269.0 (M^^•^+^, 69), 88.0 (99), 72.0 (100).

*O*-(5-Formyl-3-methoxy-[1,1′-biphenyl]-2-yl) dimethylcarbamothioate (**17**). The reaction mixture was poured onto water (15 mL) cooled on an ice bath and acidified with HCl 6 M. The product was collected by filtration under reduced pressure and washed with water. η = 75%. ^1^H NMR (CDCl_3_-d_1_): δ (ppm) = 3.17 (*s*, 3H, NCH_3_), 3.34 (*s*, 3H, NCH_3_), 3.94 (*s*, 3H, OC-H_3_), 7.39 (*m*, 3H, H3′, H4′, H5′), 7.52 (*m*, 4H, H2, H6, H2′, H6′), 9.98 (*s*, 1H, CHO). ^13^C NMR (CDCl_3_-*d_1_*): δ (ppm) = 38.7 (NCH_3_), 43.3 (NCH_3_), 56.4 (OCH_3_), 109.7 (C2), 126.6 (C6), 128.0 (C4′), 128.2 (C2′, C6′), 129.1 (C3′, C5′), 134.5 (C5), 136.4 (C1), 137.2 (C1′), 145.0 (C4), 153.1 (C3), 186.4 (CSO), 191.2 (CHO). EI/MS *m*/*z* (%): 315.1 (M^^•^+^, 38), 243.0 (29), 88.0 (100), 72.0 (89).

##### III. Microwave-Assisted Newman-Kwart Rearrangement

*O*-phenyl-*N,N*-dimethylthiocarbamates **15**–**17** were placed onto a 5 mL microwave glass vial. The vial was sealed with a septum and the content was maintained under an argon atmosphere for 30 min at room temperature. After the addition of diphenyl ether, the vial was placed into the microwave cavity. The reaction took place under microwave irradiation at 240 °C for 20 min.

*S*-(4-Formyl-2-methoxyphenyl) dimethylcarbamothioate (**18**). Synthesis and structural analysis are described in the literature [24].

*S*-(4-Formyl-2,6-dimethoxyphenyl) dimethylcarbamothioate (**19**). Compound **19** was isolated by flash column chromatography (ethyl acetate/dichloromethane (3:7)) and recrystallized in dichloromethane/petroleum ether. η = 91%. ^1^H NMR (CDCl_3_-*d_1_*): δ (ppm) = 3.00 (*bs*, 3H, NCH_3_), 3.18 (*bs*, 3H, NCH_3_), 3.94 (*s*, 6H, 2 × OCH_3_), 7.16 (*s,* 2H, H2, H6), 9.97 (*s*, 1H, CHO). ^13^C NMR (CDCl_3_-*d_1_*): δ (ppm) = 37.1 (2 × NCH_3_), 56.7 (2 × OCH_3_), 105.0 (C2, C6), 112.7 (C4), 138.7 (C1), 161.8 (C3, C5), 164.5 (COS), 191.5 (CHO). EI/MS *m*/*z* (%): 269.0 (M^^•^+^, 48), 72.1 (100).

*S*-(5-Formyl-3-methoxy-[1,1′-biphenyl]-2-yl) dimethylcarbamothioate (**20**). Compound **20** was isolated by flash column chromatography (ethyl acetate/dichloromethane (3:7)) and recrystallized in dichloromethane/petroleum ether. η = 83%. ^1^H NMR (DMSO-*d_6_*): δ (ppm) = 2.96 (*bs*, 6H, 2 × NCH_3_), 3.98 (*s*, 3H, OCH_3_), 7.38 (*m*, 5H, H2′, H3′, H4′, H5′, H6′), 7.46 (*d*, *J =* 1.6 Hz, 1H, H2), 7.48 (*d*, *J =* 1.6 Hz, 1H, H6), 10.01 (*s*, 1H, CHO). ^13^C NMR (DMSO-*d_6_*): δ (ppm) = 37.2 (2 × NCH_3_), 56.8 (OCH_3_), 108.2 (C2), 124.1 (C4), 125.8 (C6), 127.7 (C4′), 127.8 (C2′, C6′), 129.4 (C3′,), 137.8 (C5), 140.3 (C1), 149.6 (C1′), 161.0 (C3), 165.0 (COS), 191.7 (CHO). EI/MS *m*/*z* (%): 315.3 (M^^•^+^, 21), 243.2 (6), 200.1 (7), 171.1 (9), 84.0 (13), 72.1 (100). 

##### IV. Knoevenagel-Doebner Condensation 

In a round bottom flask with the aldehyde (1 mmol) dissolved in anhydrous pyridine (11.0 mmol), malonic acid (1.5 mmol) and piperidine (0.35 mmol) were added. The mixture was stirred at 70 °C for 4–5 h. The general procedure was adapted from Teixeira et al. [40] with some modifications.

(*E*)-3-(4-((Dimethylcarbamoyl)thio)-3-methoxyphenyl)acrylic acid (**21**). Synthesis and structural analysis are described in literature [24].

(*E*)-3-(4-((Dimethylcarbamoyl)thio)-3,5-dimethoxyphenyl)acrylic acid (**22**). Once the reaction was complete, the reaction mixture was poured onto water (15 mL), cooled on an ice bath and acidified with HCl 6 M. The product was collected by filtration under reduced pressure and washed with water. After suspension in methanol, the solid was centrifuged at 3000 *g* for 20 min. The supernatant was discarded, yielding the purified product. η = 88%. ^1^H NMR (DMSO-*d_6_*)*:* δ (ppm) = 2.88 (*bs*, 3H, NCH_3_), 3.06 (*bs*, 3H, NCH_3_), 3.80 (*s*, 6H, 2 × OCH_3_), 6.71 (*d*, *J* = 16.0 Hz, 1H, Hα), 7.05 (*s*, *2*H, H2, H6), 7.59 (*d, J* = 16.0 Hz, 1H, Hβ), 12.46 (*bs*, 1H, COOH). ^13^C NMR (DMSO-*d_6_*)*:* δ (ppm) = 36.9 (2 × NCH_3_), 56.8 (2 × OCH_3_), 104.7 (C2, C6), 107.0 (C4), 121.3 (Cα), 138.0 (C1), 144.2 (Cβ), 161.6 (C3, C5), 164.6 (COS), 168.0 (COOH). EI/MS *m*/*z* (%): 311.1 (M^^•^+^, 40), 84.0 (9), 72.1 (100).

(*E*)-3-(6-((Dimethylcarbamoyl)thio)-5-methoxy-[1,1′-biphenyl]-3-yl)acrylic acid (**23**). Once the reaction was complete, the reaction mixture was poured onto water (15 mL), cooled on an ice bath, and acidified with HCl 6 M. The product was collected by filtration under reduced pressure and washed with water. After suspension in methanol, the solid was centrifuged at 3000 *g* for 20 min. The supernatant was discarded, yielding the purified product. η *=* 59%. ^1^H NMR (DMSO*-d_6_*)*:* δ (ppm) = 2.88 (*bs*, 6H, 2 × NCH_3_), 3.87 (*s*, 3H, OCH_3_), 6.72 (*d*, *J* = 16.0 Hz, 1H, Hα), 7.24 (*d*, *J* = 1.5 Hz, 1H, H2), 7.28 (*m*, 2H, H2′, H6′), 7.39 (*m*, 3H, H3′, H4′, H5′), 7.45 (*d*, *J =* 1.6 Hz, 1H, H6), 7.63 (*d*, *J =* 16.0 Hz, 1H, Hβ), 12.46 (*bs*, 1H, COOH). ^13^C NMR (DMSO*-d_6_*)*:* δ (ppm) = 36.9 (2 × NCH_3_), 57.0 (OCH_3_), 110.0 (C2), 118.3 (C4), 121.5 (Cα), 123.1 (C6), 127.9 (C4′), 128.2 (C2′, C6′), 129.0 (C3′, C5′), 137.2 (C5), 140.9 (C1), 143.6 (Cβ), 149.0 (C1′), 161.1 (C3), 165.0 (COS), 167.9 (COOH). EI/MS *m*/*z* (%): 357.3 (M^^•^+^, 46), 268.2 (28), 72.1 (100).

##### V. PyBOP-Mediated Cinnamic Acid Amidation

In a round bottom flask, compound 21 (1 mmol) was dissolved in DMF and DIPEA (1.01 mmol). The mixture was cooled at 0 °C on an ice bath, and a solution of PyBOP (1.01 mmol) in dichloromethane (2.25 mL) was added slowly. The mixture was stirred for 30 min, and the amine (1.01 mmol) was added. The general procedure was adapted from Gaspar et al. [41] with some modifications.

(*E*)-*S*-(4-(3-(Benzylamino)-3-oxoprop-1-en-1-yl)-2-methoxyphenyl) dimethylcarbamothioate (**24**). Flash column chromatography was performed in ethyl acetate/dichloromethane (3:7). Recrystallization in dichloromethane/petroleum ether yielded a white solid. η = 95%. ^1^H NMR (CDCl_3_ *-d_1_*): δ (ppm) = 2.90 (*bs*, 3H, NCH_3_), 3.10 *bs*, 3H, NCH_3_), 3.86 (*s*, 3H, OCH_3_), 4.53 (*d*, *J* = 5.7 Hz, 2H, NHCH_2_Ph), 6.24 (*d*, *J* = 15.6 Hz, 1H, Hα), 6.44 (*t*, *J* = 5.5 Hz, 1H, CONH), 6.96 (*d*, *J* = 1.5 Hz, 1H, H2), 7.04 (*dd*, *J* = 1.5 Hz, 7.9 Hz, 1H, H6), 7.30 (m, 5H, H2′, H3′, H4′, H5′, H6′), 7.39 (*d*, *J* = 7.9 Hz, 1H, H5), 7.53 (*d, J* = 15.6 Hz, 1H, Hβ). ^13^C NMR (CDCl_3_*-d_1_*): δ (ppm) = 37.0 (2 × NCH_3_), 43.9 (NHCH_2_Ph), 56.1 (OCH_3_), 110.9 (C2), 118.1 (C4), 120.2 (Cα), 122.0 (C6), 127.4 (C4′), 128.0 (C2′, C6′), 128.6 (C3′, C5′), 138.0 (C5) 138.3 (C1), 138.4 (C1′), 140.03 (Cβ), 160.1 (C3), 165.5 (COS), 166.3 (CONH). EI/MS *m*/*z* (%): 370.3 (M^^•^+^, 59), 281.2 (22), 91.1 (32), 72.1 (100).

(*E*)-*S*-(2-Methoxy-4-(3-oxo-3-(phenethylamino)prop-1-en-1-yl)phenyl) dimethylcarbamothioate (**25**). Flash column chromatography was performed in ethyl acetate/dichloromethane (3:7). Recrystallization in dichloromethane/petroleum ether yielded a white solid. η = 80%. ^1^H NMR (CDCl_3_*-d_1_*): δ (ppm) = 2.87 (*t*, *J* = 7.0 Hz, 2H, NHCH_2_CH_2_Ph), 3.00 (*bs*, 3H, NCH_3_), 3.13 (*bs*, 3H, NCH_3_), 3.62 (*m*, 2H, NHCH_2_CH_2_Ph), 3.86 (*s*, 3H, OCH_3_), 5.93 (*t, J* = 5.7 Hz, 1H, NH), 6.22 (*d*, *J* = 15.6 Hz, 1H, Hα), 6.98 (*d, J* = 1.5 Hz, 1H, H2), 7.06 (*dd*, *J* = 1.5 Hz, 7.9 Hz, 1H, H6), 7.28 (*m*, 5H, H2′, H3′, H4′, H5′, H6′), 7.42 (*d, J* = 7.9 Hz, 1H, H5), 7.52 (*d, J* = 15.6 Hz, 1H, Hβ). ^13^C NMR (CDCl_3_*-d_1_*): δ (ppm) = 35.0 (NHCH_2_CH_2_Ph), 37.0 (2 × NCH_3_), 40.8 (NHCH_2_CH_2_Ph), 56.1 (OCH_3_), 110.8 (C2), 118.3 (C4), 120.1 (Cα), 122.1 (C6), 126.5 (C4′), 128.7 (C2′, C6′), 128.8 (C3′, C5′), 138.1 (C5) 138.25 (C1), 139.0 (C1′), 140.0 (Cβ), 160.1 (C3), 165.6 (COS), 166.1 (CONH). EI/MS *m*/*z* (%): 384.3 (M^^•^+^, 47), 295.2 (13), 105.1 (16), 72.1 (100).

##### VI. Alkaline Hydrolysis 

To a solution of the *S*-phenyl-*N,N*-dimethylthiocarbamate (1 mmol) in methanol (1.5 mL), aqueous solution of sodium hydroxide 2 M (5–17.5 mmol) was added dropwise. The reaction mixture was stirred at 90 °C until the TLC analysis showed maximal substrate consumption.

(*E*)-3-(4-Sulfanyl-3-methoxyphenyl)acrylic acid (TFA, **2**). Synthesis and structural analysis is described in literature [24].

(*E*)-*N*-Benzyl-3-(4-sulfanyl-3-methoxyphenyl)acrylamide (**3**). Compound **3** was obtained by an alkaline hydrolysis of compound **24** with the following conditions: compound **24** (0.82 g, 2.20 mmol), methanol (4.5 mL), and aqueous solution of NaOH 2 M (5.50 mL, 11.0 mmol). The mixture was protected from the light and stirred at 90 °C for 1.5 h. Upon completion, the mixture was allowed to cool at room temperature, then poured onto cooled aqueous saturated solution of NH_4_Cl (15 mL) and acidified with aqueous solution of acetic acid 5 M added dropwise. The solid was filtered under reduced pressure and washed with water. TLC analysis was performed in ethyl acetate/dichloromethane/formic acid (3:7:0.01). η = 53%. ^1^H NMR (MeOD-*d_4_*): δ (ppm) = 3.91 (*s*, 3H, OCH_3_), 4.49 (*s*, 2H, NHCH_2_Ph), 6.60 (*d*, *J* = 15.7 Hz, 1H, Hα), 7.05 (*dd*, *J* = 1.5 Hz, 8.0 Hz, 1H, H6), 7.10 (*d*, *J* = 1.3 Hz, 1H, H2), 7.24 (*m*, 2H, H5 H4′), 7.32 (*m*, 4H, H2′, H3′, H5′, H6′), 7.51 (*d, J* = 15.7 Hz, 1H, Hβ). ^13^C NMR (MeOD-*d_4_*): δ (ppm) = 42.9 (NHCH_2_Ph), 55.0 (OCH_3_), 109.2 (C2), 119.4 (Cα), 120.5 (C6), 124.3 (C4), 126.9 (C4′), 127.2 (C2′, C6′), 128.2 (C3′, C5′), 128.7 (C5) 133.0 (C1), 138.5 (C1′), 140.4 (Cβ), 155.1 (C3), 167.2 (CONH). ESI/MS *m*/*z* (%): 300 (M^+^ + 1, 100), 284 (55).

(*E*)-3-(4-Sulfanyl-3-methoxyphenyl)-*N*-phenethylacrylamide (**4**). Compound **4** was obtained by an alkaline hydrolysis of compound **25** with the following conditions: compound **25** (0.42 g, 1.1 mmol), methanol (1.6 mL), and an aqueous solution of NaOH 2 M (2.7 mL, 5.5 mmol). The mixture was protected from the light and stirred at 90 °C for 1.5 h. Upon completion, the mixture was allowed to cool at room temperature, then poured onto a cooled aqueous saturated solution of NH_4_Cl (15 mL) and acidified with aqueous solution of acetic acid 5 M added dropwise. The solid was filtered under reduced pressure and washed with water. TLC analysis was performed in ethyl acetate/dichloromethane/formic acid (3:7:0.01). η = 45%. ^1^H NMR (CDCl_3_-*d_1_*): δ (ppm) = 2.89 (*t*, *J* = 7.0 Hz, 2H, NHCH_2_CH_2_Ph), 3.66 (*m*, 2H, NHCH_2_CH_2_Ph), 3.90 (*s*, 3H, OCH_3_), 5.66 (*m*, 1H, NH), 6.29 (*d*, *J* = 15.5 Hz, 1H, Hα), 6.92 (*d, J* = 1.5 Hz, 1H, H2), 6.99 (*dd*, *J* = 1.5 Hz, 8.0 Hz, 1H, H6), 7.23 (*m*, 4H, H5, H3′, H4′, H5′), 7.32 (*m*, H2′, H6′), 7.53 (*d, J* = 15.5 Hz, 1H, Hβ). ^13^C NMR (CDCl_3_-*d_1_*): δ (ppm) = 35.7 (NHCH_2_CH_2_Ph), 40.8 (NHCH_2_CH_2_Ph), 55.9 (OCH_3_), 109.5 (C2), 119.9 (Cα), 120.8 (C6), 123.4 (C4), 126.6 (C4′), 128.7 (C2′, C6′), 128.8 (C3′, C5′), 129.1 (C5), 133.2 (C1), 138.9 (C1′), 140.7 (Cβ), 154.8 (C3), 165.9 (CONH). ESI/MS *m*/*z* (%): 314 (M^+^ + 1, 100). 

(*E*)-3-(4-Sulfanyl-3,5-dimethoxyphenyl)acrylic acid (**5**). Compound **5** was obtained by an alkaline hydrolysis of compound **22** with the following conditions: compound **22** (0.64 g, 2.05 mmol), methanol (2 mL) and aqueous solution of NaOH 2 M (12.5 mL, 2.5 mmol). The mixture was protected from the light and stirred at 90 °C for 9 h. Upon completion, the mixture was allowed to cool at room temperature, then poured onto a cooled aqueous saturated solution of NH_4_Cl (15 mL) and acidified with aqueous solution of acetic acid 5 M added dropwise. The solid was filtered under reduced pressure and washed with water. TLC analysis was performed in ethyl acetate/dichloromethane/formic acid (3:7:0.01). η = 79.6%. ^1^H NMR (CDCl_3_-*d_1_*)*:* δ (ppm) = 3.94 (*s*, 6H, 2 × OCH_3_), 4.19 (*s*, 1H, SH), 6.40 (d, *J* = 15.9 Hz, 1H, Hα), 6.74 (*s*, *2*H, H2, H6), 7.72 (*d, J* = 16.0 Hz, 1H, Hβ). ^13^C NMR (CDCl_3_-*d_1_*)*:* δ (ppm) = 56.3 (2 × OCH_3_), 103.8 (C2, C6), 114.2 (C4), 116.3 (Cα), 131.0 (C1), 147.1 (Cβ), 155.4 (C3, C5), 172.2 (COOH). ESI/MS *m*/*z* (%): 241 (M^+^ + 1, 100).

(*E*)-3-(6-Sulfanyl-5-methoxy-[1,1′-biphenyl]-3-yl)acrylic acid (**6**). Compound **6** was obtained by an alkaline hydrolysis of compound **23** the following conditions: compound **23** (0.36 g, 1.01 mmol), methanol (2 mL) and aqueous solution of NaOH 2 M (8.75 mL, 17.5 mmol). The mixture was protected from the light and stirred at 90 °C for 16 h. Upon completion, the mixture was allowed to cool at room temperature, then poured onto cooled aqueous saturated solution of NH_4_Cl (15 mL) and acidified with aqueous solution of acetic acid 5 M added dropwise. The solid was filtered under reduced pressure and washed with water. TLC analysis was performed in ethyl acetate/dichloromethane/formic acid (3:7:0.01). η = 73%. ^1^H NMR (MeOD-*d_4_*): δ (ppm) = 3.99 (*s*, 3H, OCH_3_), 6.47 (*d*, *J* = 15.9 Hz, 1H, Hα), 7.03 (*d*, *J* = 1.4 Hz, 1H, H2), 7.20 (*d*, *J* = 1.7 Hz, 1H, H6), 7.40 (*m*, 5H, H2′ H3′, H4′, H5′, H6′), 7.63 (*d*, *J =* 15.9 Hz, 1H, Hβ). ^13^C NMR (MeOD*-d_4_*): δ (ppm) = 55.6 (OCH_3_), 107.7 (C2), 117.5 (Cα), 122.6 (C6), 124.3 (C4), 127.6 (C4′), 128.1 (C2′, C6′), 128.7 (C3′, C5′), 131.5 (C5), 140.2 (C1), 141.2 (C1′), 144.4 (Cβ), 154.5 (C3), 169.2 (COOH). ESI/MS *m*/*z* (%): 287 (M^+^ + 1, 100).

### 3.2. Radical Scavenging Activity

#### 3.2.1. Spectrophotometric Methods

##### I. DPPH^•^ Radical Assay

DPPH^•^ radical scavenging activity was performed as described by Teixeira et al. [40] (see Supplementary Information).

##### II. ABTS^^•^+^ Radical Cation Assay

ABTS^^•^+^ scavenging activity was evaluated as described by Teixeira et al. [40] (see Supplementary Information).

##### III. GO^•^ Radical Assay

GO^•^ radical scavenging protocol was adapted from the literature [42,43,44] and described in Supplementary Information.

#### 3.2.2. Fluorometric Methods (ORAC-FL Assay)

The ORAC-FL analyses were performed using an experimental protocol adapted from the literature [45,46] and described in Supplementary Information.

### 3.3. Electrochemical Measurements

DPV experiments were performed as described elsewhere [23] (see Supplementary Information).

### 3.4. In Vitro Toxicology

#### 3.4.1. SH-SY5Y Cell Culture

Human SH-SY5Y neuroblastoma cells were obtained from the American Type Culture Collection (ATCC, Manassas, VA, USA). SH-SY5Y cell culture and differentiation were performed as previously described by Fernandes et al. [32] and in Supplementary Information.

#### 3.4.2. Cytotoxicity

Stock solutions of the test compounds (1, 10 and 50 mM) were freshly prepared in ethanol absolute. Final test concentrations of the compounds under study were obtained by diluting into culture medium immediately before use, giving a final maximum concentration of 0.1% ethanol.

In cytotoxicity experiments, differentiated SH-SY5Y cells were exposed to the test compounds (1, 10 and 50 µM) for 24 h. Controls were treated with culture media containing 0.1% ethanol. Cell viability was estimated using the MTT reduction assay [32] and the NR uptake assay [47] (see Supplementary Information).

#### 3.4.3. Protection against Oxidative Stress Inducers

To evaluate the ability of phenols and thiophenols to protect neuronal cells from oxidative stress, hydrogen peroxide (H_2_O_2_) and ferric nitrilotriacetate (FeNTA) were used as inducers of oxidative damage. Differentiated SH-SY5Y cells were pre-treated with the compounds under study for 24 h and exposed to the oxidative stressors.

##### I. Cytoprotective Properties against H_2_O_2_-Induced Damage

Differentiated SH-SY5Y cells were pretreated with the test compounds (10 and 50 µM) for 24 h. Then, the culture medium was removed and replaced with medium containing H_2_O_2_ 1.5 mM, and cells were incubated for additional 30 min at 37 °C in a humidified, 5% CO_2_−95% air atmosphere. Cell viability was estimated by the MTT reduction assay [32].

##### II. Cytoprotective Properties against FeNTA-Induced Damage

Nitrilotriacetic acid disodium salt solution (500 mM) and nitrilotriacetic acid trisodium salt solution (500 mM) were mixed until pH 7 was achieved and diluted to obtain a concentration of 250 mM. This solution was added to the required amount of iron(III) chloride to obtain the NTA:iron(III) molar ratio of 2.5:1 ([Fe(III) = 100 mM). Then, the ferric nitrilotriacetic acid (FeNTA) solution was diluted in culture medium to obtain a final iron(III) solution with concentration of 4 mM.

Differentiated SH-SY5Y cells were pretreated with the test compounds (10 and 50 µM) for 24 h. Then, the culture medium was removed and replaced with medium containing FeNTA 4 mM. Cells were incubated for additional 24 h at 37 °C in a humidified, 5% CO_2_−95% air atmosphere. Cell viability was estimated by the MTT reduction assay [32].

#### 3.4.4. Statistical Analysis

Data analysis for all the studies are specified in Supplementary Information.

### 3.5. Estimation of Drug-Like Properties

The calculation of molecular weight (MW), topological polar surface area (TPSA), number of hydrogen bond donors (HBD) and acceptors (HBA), number of rotatable bonds (RB) and logarithm of the ratio of the concentration of a drug in the brain and in the blood (logBB) was performed using the Stardrop software. The calculation of octanol/water partition coefficient (clogP) was performed using the SwissADME (http://swissadme.ch/index.php).

## 4. Conclusions

In this work, we successfully obtained a series of TFA and FA derivatives, and evaluated their antioxidant activity, as well as their cytotoxicity and cytoprotective profiles. Structural diversity was attained by incorporating 5-methoxy or 5-phenyl groups at the aromatic ring, or benzyl and phenethyl amide groups at the carboxylic acid group.

Thiophenols displayed remarkable radical scavenging properties. Compared to phenols, thiophenols reacted faster and more efficiently with DPPH^•^ and GO^•^ radicals, and presented lower *E*_p_ values at physiological pH. However, unlike phenols, thiophenols were unable to scavenge ROO^•^ radicals in ORAC-FL assay. The presence of different substituents directly attached to the aromatic ring did not improve the antioxidant profile of the parent compounds (TFA and FA). Although a slight decrease of *E*_p_ values was observed, 5-methoxy and 5-phenyl derivatives showed similar or lower radical scavenging activity, and slower reaction rates than the unsubstituted analogues. On the other hand, the carboxylic acid amidation was proven to be a beneficial structural modification of the cinnamic acid scaffold, since it preserved the antioxidant profile of the parent acids while improving the drug-like properties of the antioxidants. The cytotoxicity of FA and TFA derivatives in differentiated SH-SY5Y cells was similar to that of the parent compounds at concentrations up to 50 µM. Among the tested compounds, only TFA **2**, TFA benzyl amide **3**, and 5-phenylferulic acid **10** attenuated the oxidative damage inflicted by H_2_O_2_ and iron overload. Compound **3** is highlighted as the best antioxidant of the series with favorable physicochemical properties.

Overall, the results obtained in this work emphasized the dissimilar biological outline of thiophenols and phenols, as well as the effect of the structural modification of FA and TFA on their antioxidant properties. The data may be useful for the development of new drugs able to tackle the events that lead to the progression of neurodegenerative disorders.

## Figures and Tables

**Table 1:** Radical scavenging activities and redox potentials (*E*_p_) of thiophenols **2**–**6** and phenols **1**, **7**–**10**. The results obtained in DPPH^•^, ABTS^^•^+^, GO^•^ assays are expressed as mean IC_50_ standard deviation (*n* = 3).

Compound	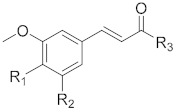			IC_50_ (µM)			ORAC-FL Index	*E*_p_ (mV)
	R_1_	R_2_	R_3_	DPPH^•^	ABTS^^•^+^	GO^•^		
**2 (TFA)**	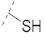	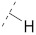	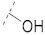	47.8 ± 0.9*^a^*	19.9 ± 1.0*^a^*	4.25 ± 0.10*^a^*	0.34 ± 0.04*^a^*	271
**3**	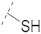	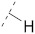	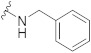	49.8 ± 0.7	20.6 ± 0.7	4.50 ± 0.08	0.12 ± 0.02	198
**4**	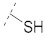	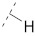	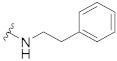	47.3 ± 0.7	19.9 ± 0.3	4.41 ± 0.15	0.15 ± 0.01	186
**5**	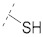	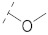	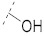	42.7 ± 2.1	22.9 ± 0.6	6.70 ± 0.07	0.30 ± 0.03	218
**6**	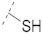	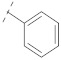	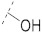	50.7 ± 1.6	27.0 ± 1.8	6.66 ± 0.13	0.34 ± 0.01	245
**1(FA)**	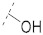	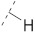	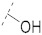	64.6 ± 1.9*^a^*	16.8 ± 0.4*^a^*	37.5 ± 2.5*^a^*	3.2 ± 0.1*^a^*	344 *^b^*
**7**	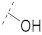	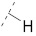	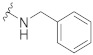	61.7 ± 0.8	25.6 ± 1.8	76.4 ± 3.6	4.0 ± 0.4	327
**8**	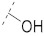	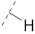	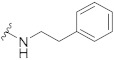	61.2 ± 0.7	26.0 ± 1.7	70.7 ± 2.5	3.7 ± 0.3	338
**9**	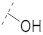	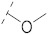	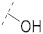	43.9 ± 1.6	16.6 ± 0.3	3.90 ± 0.1	3.2 ± 0.3	291
**10**	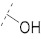	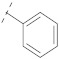	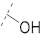	69.7 ± 3.8	19.8 ± 0.8	31.2 ± 3.9	2.7 ± 0.1	351
**Trolox**	----	----	----	24.6 ± 0.9	18.2 ± 0.5	2.98 ± 0.15	1.00 ± 0.02	110

*^a^* Data from Chavarria et al. [24]. *^b^* Data from Garrido et al. [23].

**Table 2:** Predicted physicochemical properties of TFA (compound **2**) and compounds **3** and **10**.

Compound	MW *^a^*	cLogP*^b^*	TPSA *^a^*	HBA *^a^*	HBD *^a^*	RB	LogBB
**2**	210.25	1.97	46.53	4	2	3	−0.9373
**3**	299.39	3.25	38.33	4	2	6	0.02948
**10**	270.28	2.76	66.76	4	2	4	-1.511
CNS^+^ drugs	< 500 *^c^*	2–5 *^c^*	< 90 *^c^*	< 7 *^d^*	< 3 *^d^*	< 8 *^d^*	<−1

*^a^* Properties predicted using the Stardrop software. MW: molecular weight; TPSA: topological polar surface area; HBA: number of H-bond acceptor atoms; HBD: number of H-bond donor atoms; RB: number of rotatable bonds; logBB: logarithm of the ratio of the concentration of a drug in the brain and in the blood. *^b^* Properties predicted using the SwissADME (http://swissadme.ch/index.php); clogP: logarithm of the octanol/water partition coefficient. *^c^* Data from Hitchcock et al. [37]. *^d^* Data from Pajouhesh et al. [38].

## Supplementary Materials

The following are available online, Experimental details, Statistics, Figure S1: DPPH^•^ kinetic curves of thiophenols **2**–**6** and phenols **1**, **7**–**10**; Figure S2**:** ABTS^^•^+^ kinetic curves of thiophenols **2**–**6** and phenols **1**, **7**–**10**; Figure S3**:** GO^•^ kinetic curves of thiophenols **2**–**6** and phenols **1**, **7**–**10**; Figure S4: Protection against (**A**) H_2_O_2_- and (**B**) FeNTA-induced damage in differentiated SH-SY5Y cells by caffeic acid (CA); Figure S5: ^1^H and ^13^C NMR spectra of final compound **3** (NMR spectrum obtained in MeOD-d_4_); Figure S6: ^1^H and ^13^C NMR spectra of final compound **4** (NMR spectrum obtained in MeOD-d_4_); Figure S7: ^1^H and ^13^C NMR spectra of final compound **5** (NMR spectrum obtained in MeOD-d_4_); Figure S6: ^1^H and ^13^C NMR spectra of final compound **6** (NMR spectrum obtained in MeOD-d_4_).
