# Supplementary material for: Insights into the Discovery of Novel Neuroprotective Agents: A Comparative Study between Sulfanylcinnamic Acid Derivatives and Related Phenolic Analogues
Source: Molecules. 2019 Dec 2;24(23):4405. doi: 10.3390/molecules24234405 (PMC6930627; doi:10.3390/molecules24234405)
Supplement: Supplementary file 1 [file molecules-24-04405-s001.pdf]

Electronic Supplementary Information

Insights into the discovery of novel neuroprotective agents: a comparative study between sulfanylcinnamic acid derivatives and related phenolic analogues

Daniel Chavarria[1,2‡], Carlos Fernandes[1‡], Brandon Aguiar[1], Tiago Silva[1,2], Jorge Garrido[3], Fernando Remião[4], Paulo J. Oliveira[2], Fernanda Borges[1*]

[1] CIQUP/Department of Chemistry and Biochemistry, Faculty of Sciences, University of Porto, 4169-007 Porto, Portugal

[2] CNC - Center for Neuroscience and Cell Biology, University of Coimbra, UC Biotech, Biocant Park, Cantanhede, Portugal.

[3] Department of Chemical Engineering, School of Engineering (ISEP), Polytechnic of Porto, 4200-072 Porto, Portugal

[4] UCIBIO-REQUIMTE, Laboratory of Toxicology, Department of Biological Sciences, Faculty of Pharmacy, University of Porto, Porto, Portugal

‡ These authors contributed equally to this work.

* Corresponding Authors:

Fernanda Borges, CIQUP/Department of Chemistry and Biochemistry, Faculty of Sciences, University of Porto, Porto 4169-007, Portugal. E-mail: fborges@fc.up.pt

Contents of Electronic Supporting information:

1. Experimental section 3

1.1. Chemistry 3

1.1.1. Reagents 3

1.1.2. Materials and apparatus. 3

1.2. Radical scavenging activity 3

1.2.1. Spectrophotometric methods 3

1.2.1.1. DPPH$^{\bullet}$ radical assay 4

1.2.1.2. ABTS$^{\bullet+}$ radical cation assay 4

1.2.1.3. GO$^{\bullet}$ radical assay 4

1.2.2. Fluorometric methods (ORAC-FL assay) 4

1.3. Electrochemical measurements 5

1.4. *In vitro* toxicology 6

1.4.1. Materials 6

1.4.2. SH-SY5Y cell culture 6

1.4.3. Cytotoxicity assays 6

1.4.3.1. MTT reduction assay 6

1.4.3.2. NR uptake assay 7

1.4.4. Statistical analysis 7

2. Additional figures 8

3. References 17

1. Experimental section

1.1. Chemistry

1.1.1. Reagents

Ferulic acid, vanillin, 5-bromovanillin, syringaldehyde, phenylboronic acid, potassium carbonate (K_2CO_3), tetrabutylammonium bromide (TBAB), diazabicyclo[2.2.2]octane (DABCO), dimethylthiocarbamoyl chloride (DMTCl), malonic acid, piperidine, sodium hydroxide, diisopropylethylamine (DIPEA), (Benzotriazol-1-yloxy)tripyrrolidinophosphonium hexafluorophosphate (PyBOP), benzylamine, phenethylamine, *N,N*-dimethylformamide (DMF), diphenyl ether, anhydrous pyridine, methanol and dichloromethane, were purchased from Sigma Aldrich, Alfa-Aesar or TCI. All solvents were *pro analysis* grade from Merck, Carlo Erba Reagents and Scharlab. Deionized water (conductivity < 0.1 $mS.cm^{-1}$) was used throughout all the experiments.

1.1.2. Materials and apparatus.

Microwave-assisted synthesis was performed in a Biotage® Initiator Microwave Synthesizer. The monitoring of reactions progress was performed by thin layer chromatography (TLC) on precoated silica gel 60 F254 acquired from Merck (Darmstad, Germany). TLC spots were visualized under UV detection (254 nm and 365 nm). After the extraction step, organic layers were washed with brine, dried over anhydrous sodium sulphate and concentrated under reduced pressure. Some crude products were purified by flash column chromatography. Flash column chromatography was carried out on silica gel 60 Å acquired from Carlo-Erba Reactifs (SDS, France). The fractions with the desired compound were gathered and concentrated *in vacuo*. The solvents were evaporated using a Buchi Rotavapor.

^{1}H and ^{13}C NMR and DEPT135 data were acquired, at room temperature, on a Brüker AMX 300 spectrometer operating at 400 and 100 MHz, respectively. The solvents used in NMR experiments included $CDCl_3$-d_1, MeOD-d_4, and DMSO-d_6. The chemical shifts are expressed in δ (ppm) values relative to tetramethylsilane (TMS) as internal reference and coupling constants (*J*) are given in Hz. Carbon signals present in DEPT135 spectra were underlined. Electron impact mass spectrometry (EI-MS) spectra were obtained on a Varian 320-MS apparatus. Electrospray ionization mass spectrometry (ESI-MS) spectra were obtained on an LTQ Orbitrap™ XL hybrid mass spectrometer (Thermo Fischer Scientific, Bremen, Germany) controlled by *LTQ Tune Plus 2.5.5* and *Xcalibur 2.1.0*. The data are reported as m/z (% of relative intensity of the most important fragments).

1.2. Radical scavenging activity

1.2.1. Spectrophotometric methods

To measure the radical scavenging activity of the test compounds, $DPPH^{\bullet}$, $ABTS^{\bullet+}$ and $GO^{\bullet}$ assays were adopted. Sodium persulfate, $DPPH^{\bullet}$, 2,2′-azino-bis(3-ethylbenzothiazoline-6-sulfonic acid) diammonium salt (ABTS) and $GO^{\bullet}$ were acquired from Sigma Aldrich. In these methods, the scavenging of the colored radical by an antioxidant is recorded by spectrophotometry. The absorbance decrease was monitored in a multiplate reader (BioTek Synergy HT from BioTek Instruments, Winooski, VT, USA). The results were expressed in IC_{50} as mean ± standard deviation (n = 3). Trolox (Sigma Aldrich), a water-

soluble vitamin E derivative, was used as a standard reference. Kinetic curves were prepared using GraphPad PRISM version 6 for Windows (GraphPad Software®, San Diego, CA, USA).

1.2.1.1. DPPH$^{\bullet}$ radical assay

DPPH$^{\bullet}$ radical scavenging activity was performed as described by Teixeira *et al.*[1] Briefly, solutions of the test compounds with increasing concentrations (ranging between 10 μM and 1 mM) were prepared in ethanol. A DPPH$^{\bullet}$ ethanolic solution (6.85 μM) was also prepared and then diluted to reach the absorbance of 0.72 ± 0.02 at 515 nm. After the addition of DPPH$^{\bullet}$ solution (180 μL) to the compound solution (20 μL) in triplicate, the absorbance at 515 nm was recorded every minute over 45 min. The percent inhibition of the radical was based on the comparison between the blank (20 μL of ethanol and 180 μL of DPPH$^{\bullet}$ solution), which corresponds to 100% of the radical, and the test compound solutions. The dose–response curves allowed the determination of IC_{50} values.

1.2.1.2. ABTS$^{\bullet+}$ radical cation assay

ABTS$^{\bullet+}$ scavenging activity was evaluated as described by Teixeira *et al.* [1] Briefly, ethanolic solutions of the test compounds with increasing concentrations (ranging between 50 μM and 500 μM) were prepared. ABTS$^{\bullet+}$ radical cation solution was obtained by the addition of aqueous solution of potassium persulfate (150 mM, 163 μL) to the aqueous solution of ABTS (7 mM, 10 mL) and the storage in the dark at room temperature for 16 h (2.45 mM final concentration). The solution was then diluted in ethanol to reach the absorbance of 0.72 ± 0.02 at 734 nm. After the addition of ABTS$^{\bullet+}$ solution (180 μL) to the compound solution (20 μL) in triplicate, the spectrophotometric measurement was carried out every minute over 15 min at 734 nm. The percent inhibition of the radical was based on the comparison between the blank (20 μL of ethanol and 180 μL of ABTS$^{\bullet+}$ solution), which corresponds to 100% of radical, and the test compound solutions. The dose–response curves allowed the determination of IC_{50} values.

1.2.1.3. GO$^{\bullet}$ radical assay

GO$^{\bullet}$ radical scavenging protocol was adapted from the literature [2-4]. Solutions of test compounds with increasing concentrations (ranging between 5 μM to 1 mM) were prepared in ethanol. An ethanolic solution of GO$^{\bullet}$ 5 mM was prepared and diluted to reach the absorbance of 1.00 ± 0.02 at 428 nm. After the addition in triplicate of compound solution (20 μL) to GO$^{\bullet}$ solution (180 μL), the spectrophotometric measurement was performed at 428 nm over 30 min. The percent inhibition of radical was based on comparison between the blank (20 μL of ethanol and 180 μL of GO$^{\bullet}$ solution), which corresponds to 100 % of response, and test compounds solutions. The dose-response curves allowed the determination of IC_{50} values.

1.2.2. Fluorometric methods (ORAC-FL assay)

The ORAC-FL analyses were performed on a multiplate reader (BioTek Synergy HT from BioTek Instruments, Winooski, VT, USA) using black polystyrene 96-well plates. Fluorescence was measured from

the top, at an excitation wavelength of 485/20 nm and an emission at 528/20 nm. The plate reader was controlled by Gen5 software. 2,2'-Azobis-(amidinopropane) dihydrochloride (AAPH), (±)-6-hydroxy-2,5,7,8-tetramethylchromane-2-carboxylic acid (trolox) and fluorescein (FL) were purchased from Sigma-Aldrich. The general procedure was adapted from the literature [5, 6], with some modifications.

The reaction was carried out in 75 mM of sodium phosphate buffer solution (pH = 7.4) at a final volume of 200 µL. Stock solutions of FL (5 mM) and antioxidants (5 mM) were prepared in ethanol due to their low solubility in buffer at high concentrations. Then, they were diluted in sodium phosphate buffer solution (pH = 7.4). The range of antioxidant concentrations was empirically selected to obtain a good separation between the fluorescence curves. The test compounds (final concentrations range of 0.1-2 µM) and fluorescein (40 nM, final concentration) were placed in each well and pre-incubated for 15 min at 37 ℃ before the addition of AAPH (final concentration 18 mM). The microplate was immediately placed in the reader and automatically shaken prior to each reading. The fluorescence was measured minutely for 120 min. A control assay with fluorescein, AAPH and buffer (instead of the antioxidant solution) was performed for each assay. Trolox was also included in each assay as standard antioxidant in a final concentration range of 2-8 µM. All reaction mixtures were prepared in triplicate and at least three independent experiments were performed for each test compound. The fluorescence decay curves were normalized by the maximum value of fluorescence, and the areas under the curves (AUC) were determined according to the following **Equation 1**.

$$\mathrm{AUC} = 1 + \sum_{i=1}^{i=120} \frac{f_i}{f_0} = 1 + \frac{f_1}{f_0} + \frac{f_2}{f_0} + \frac{f_3}{f_0} + \ldots + \frac{f_{120}}{f_0} \quad \textbf{(Equation 1)}$$

where f_0 = initial fluorescence reading at 0 min and f_i = fluorescence reading at time i. The net AUC is obtained by subtracting the AUC of the blank from that of a sample. The inhibition capacity, expressed as the relative Trolox equivalent ORAC values, was quantified employing the **Equation 2**.

$$\text{relative ORAC value} = \frac{\mathrm{AUC}_{\mathrm{antioxidant}} - \mathrm{AUC}_{\mathrm{control}}}{\mathrm{AUC}_{\mathrm{trolox}} - \mathrm{AUC}_{\mathrm{control}}} \times \frac{[\mathrm{Trolox}]}{[\mathrm{antioxidant}]} \quad \textbf{(Equation 2)}$$

where $AUC_{control}$ = area under the curve of control; $AUC_{antioxidant}$ = area under the curve of the test compound; AUC_{trolox} = area under the curve of Trolox (standard); [trolox] = Trolox concentration; and [antioxidant] = antioxidant concentration.

1.3. Electrochemical measurements

Voltammetric studies were carried out using an Autolab PGSTAT 12 potentiostat/galvanostat (Metrohm-Autolab, Netherlands) and one compartment glass electrochemical cell. The voltammetric data was acquired at room temperature using a three-electrode system: a glassy carbon working electrode (GCE) (d = 2 mm), a platinum wire counter electrode and an Ag/AgCl saturated KCl reference electrode (Metrohm, Switzerland). The working electrode was polished manually with an aqueous slurry of alumina powder (BDH Chemicals, VWR, USA) on a microcloth pad and rinsed with water before use. A Crison pH-meter with glass electrode was used for the pH measurements (Crison, Barcelona, Spain).

Voltammetric measurements were performed as described elsewhere [7]. Test solutions were prepared directly in the electrochemical cell, by dilution of ethanolic solutions of the compounds under

study (10 mM, 100 μL) in the supporting electrolyte (10 mL) to obtain a final concentration of 100 μM. The scan rates used in differential pulse voltammetry (DPV) was 5 mV.s^{-1}.

1.4. *In vitro* toxicology

1.4.1. Materials

All reagents used were of analytical grade or of the highest grade available. Neutral red (NR) solution, 3-(4,5-dimethylthiazol-2-yl)-2,5-diphenyltetrazolium (MTT) bromide, Dulbecco's Modified Eagle's Medium (DMEM) with 4.5 g/L glucose, retinoic acid, 12-*O*-tetradecanoylphorbol-13-acetate (TPA), ascorbic acid, hydrogen peroxide (H_2O_2) solution 30 % (w/w), iron(III) chloride and nitrilotriacetic acid disodium salt were obtained from Sigma Aldrich (St. Louis, MO, USA). Nitrilotriacetic acid trisodium salt was obtained from Alfa Aesar (Karlsruhe, Germany). Reagents used in cell culture such as heat-inactivated fetal bovine serum (FBS), antibiotic (10,000 U/mL penicillin, 10,000 μg/mL streptomycin), MEM Non-Essential Amino Acids solution (100×) (MEM NEAA), 0.25% trypsin/1 mM ethylenediamine tetraacetic acid (EDTA) and Hanks' balanced salt solution (HBSS) were purchased from Gibco Laboratories (Lenexa, KS, USA). Dimethylsulfoxide (DMSO), ethanol absolute, and acetic acid were obtained from Merck (Darmstadt, Germany).

1.4.2. SH-SY5Y cell culture

Human neuroblastoma SH-SY5Y cells were routinely cultured into 75 cm^3 flasks and maintained in DMEM with 4.5 g/L glucose supplemented with 10 % heat-inactivated FBS (v/v), 1 % MEM NEAA (v/v) and 1% penicillin/streptomycin (v/v). Cells were maintained at 37 ℃, in a humidified 5% CO_2–95% air atmosphere, and the culture medium was changed every 2-3 days. The cultures were passaged once a week by trypsinization (0.25 % trypsin/1 mM EDTA). To avoid phenotypic changes, the cells used for all experiments were taken between 19th and 28th passages. Cells were differentiated as described previously by Fernandes *et al.* [8]. Briefly, SH-SY5Y cells were seeded onto 96-well plates at a density of 25,000 cells/cm^2 in cell culture medium with *trans*-retinoic acid (RA) (final concentration of 10 μM) and incubated for 3 days, at 37 ℃, in a humidified 5% CO_2–95% air atmosphere. Then, the cell culture medium was removed and replaced with medium supplemented with 12-*O*-tetradecanoylphorbol-13-acetate (TPA) (final concentration 80 nM), and cells were incubated for an additional period of 3 days before being used in the assays. Stock solutions of RA (10 mM) and TPA (80 μM) were prepared in DMSO.

1.4.3. Cytotoxicity assays

1.4.3.1. MTT reduction assay

In the MTT reduction assay, the water-soluble MTT tetrazolium salt is reduced inside viable cells with active metabolism. The resulting purple colored formazan product accumulates as an insoluble precipitate inside the cells. The amount of formazan formed, presumably directly proportional to the number of viable cells, can be quantified spectrophotometrically after solubilization in an organic solvent [9, 10]. Briefly, after the incubation periods with test compounds or the oxidative stressors, the cell culture medium was removed and a solution of MTT 0.5 mg/mL in fresh culture medium was added. Cells were incubated at 37 ℃ in a humidified, 5% CO_2–95% air atmosphere for 1 h. Then, the cell culture medium

was removed, and the formed formazan crystals were dissolved in 100 % DMSO. The absorbance was measured at 550 nm in a multiwell microplate reader (PowerWave XS from BioTek Instruments, Vermont, US). The results were expressed as the percentage of MTT reduction relative to that of the control [MTT reduction (% of control)] ± standard error mean (SEM) of at least three independent experiments.

1.4.3.2. NR uptake assay

The neutral red (NR) uptake assay is based on the ability of viable cells to incorporate the supravital dye NR and retain it inside the lysosomes [11]. NR uptake assay was performed as previously described by Fernandes *et al*. [12] Briefly, after the 24 h incubation periods with the test compounds, the cell culture medium was removed and a solution of NR (50 mg/mL) in fresh cell culture medium was added. Differentiated SH-SY5Y cells were incubated for 1 h at 37 ℃ in a humidified, 5% CO_2–95% air atmosphere. Then, the cell culture medium was removed and replaced by ethanol absolute/distilled water (1:1) with 5 % acetic acid to liberate the dye from the viable cells. The absorbance was measured at 540 nm in a multiwell microplate reader (PowerWave XS from BioTek Instruments). The results were expressed as the percentage of NR uptake relative to that of the control (non-treated) cells [NR uptake (% of control)] ± standard error mean (SEM) of at least three independent experiments.

1.4.4. Statistical analysis

The data obtained are expressed as mean ± standard error mean (SEM) of at least three independent experiments. All statistical analyses were performed using GraphPad PRISM version 6 for Windows (GraphPad Software®, San Diego, CA, USA). The normality of the data distribution was evaluated using three normality tests: KS normality test, D'Agostino and Pearson omnibus normality test, and Shapiro−Wilk normality test. For data with a parametric distribution, statistical comparisons were carried out by one-way analysis of variance (ANOVA) followed by Dunnet's multiple comparison test. For data with a non-parametric distribution, statistical comparisons were estimated using the nonparametric method of Kruskal–Wallis [one-way ANOVA on ranks] followed by Dunn's *post hoc* test. In all cases, p values lower than 0.05 were considered significant.

2. Additional figures

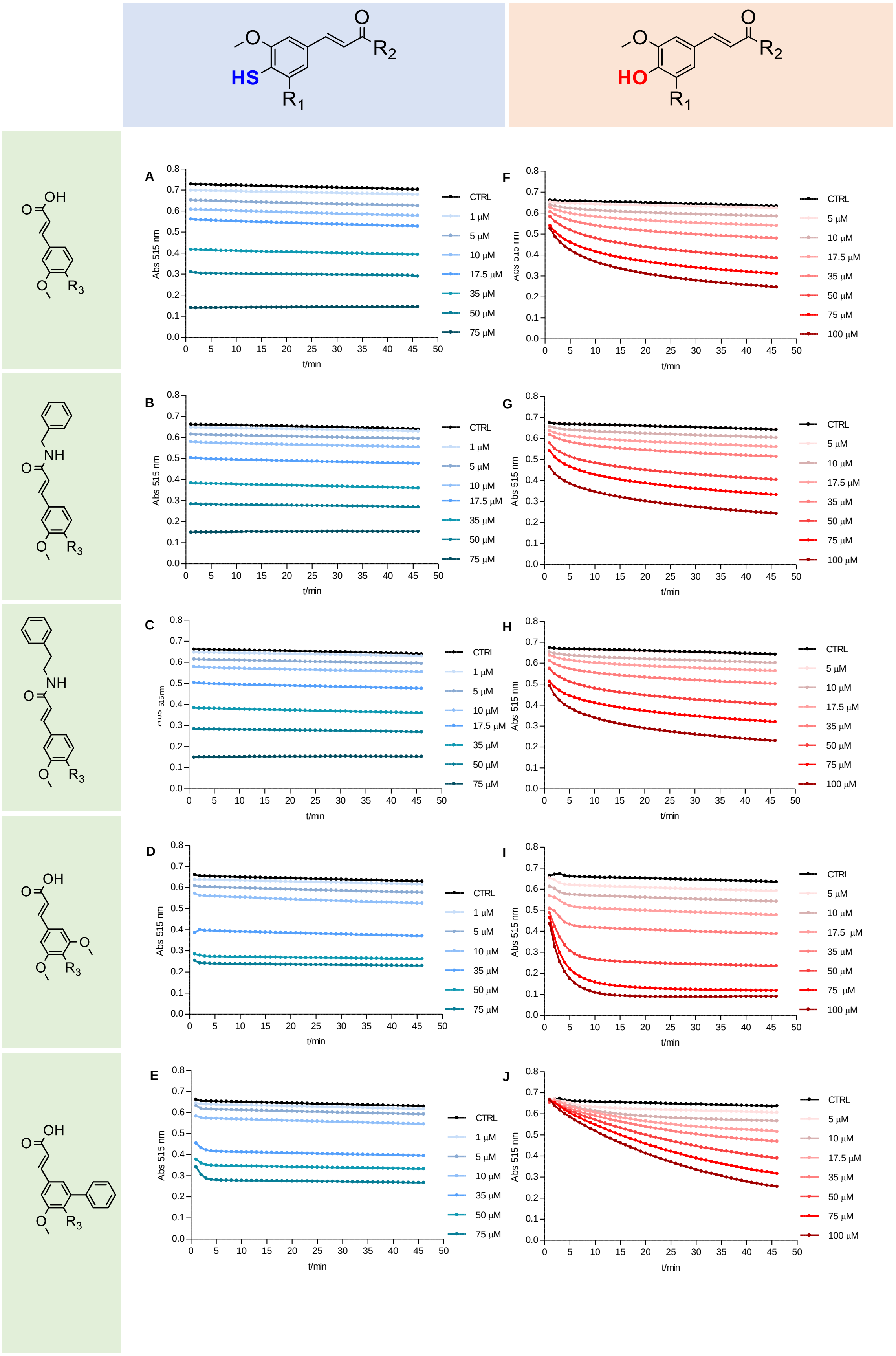

Figure S1: DPPH$^{\bullet}$ kinetic curves of thiophenols **2-6** and phenols **1**, **7-10**.

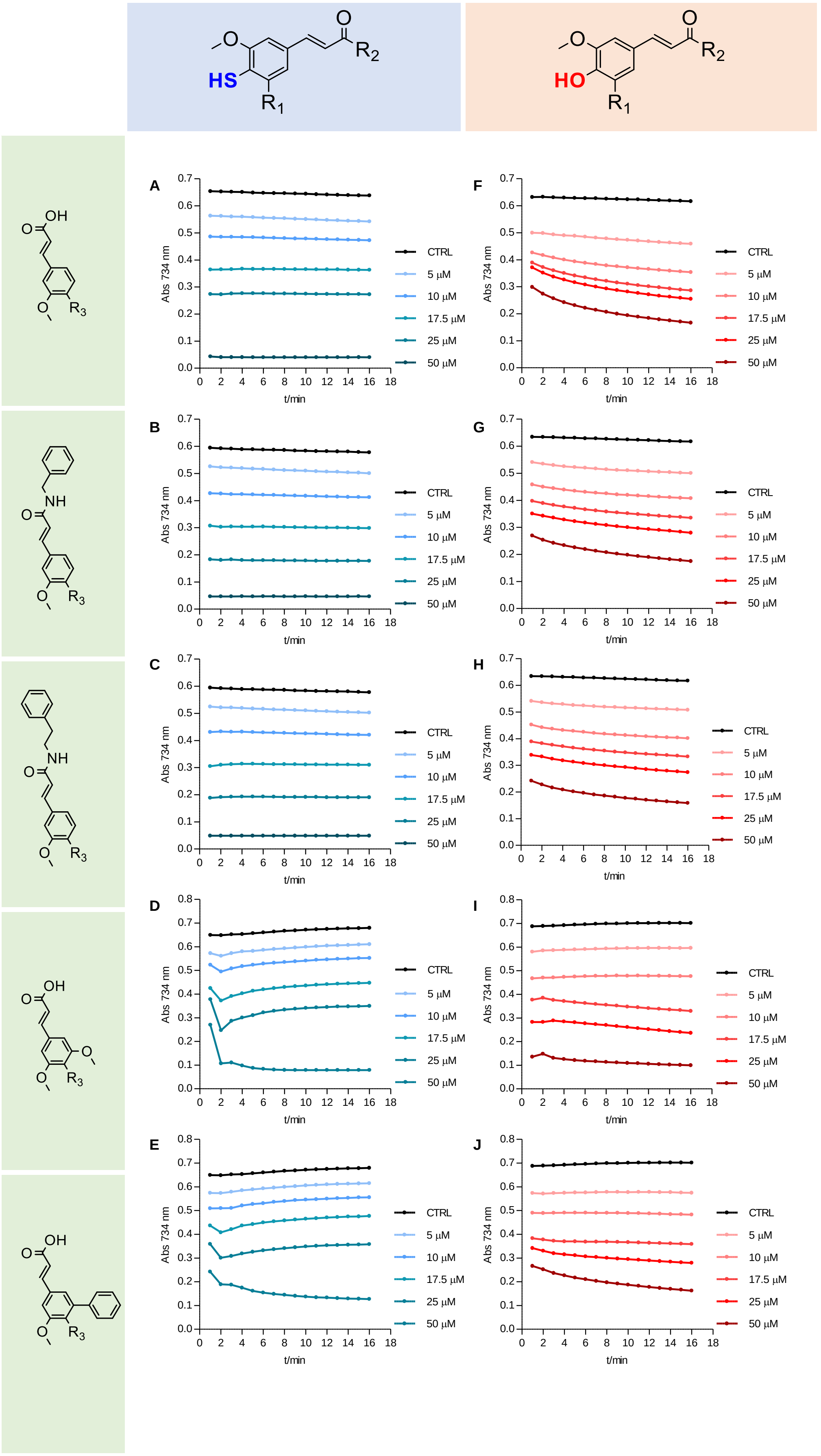

Figure S2: $ABTS^{\bullet+}$ kinetic curves of thiophenols **2-6** and phenols **1**, **7-10**.

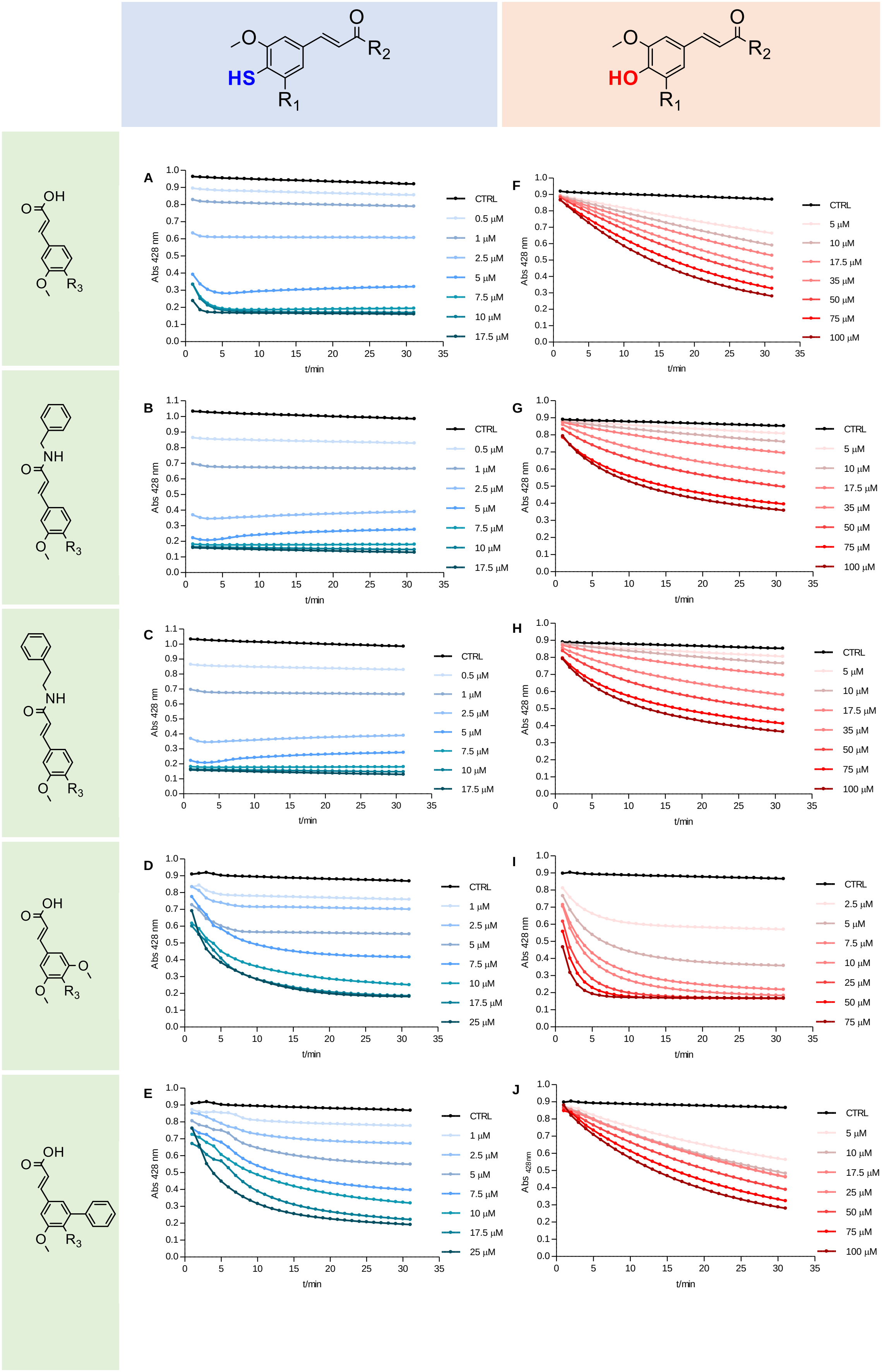

HS
HO
R1
R2
R3
OH
NH
A
B
C
D
E
F
G
H
I
J
Abs 428 nm
Abs 428nm
t/min
CTRL
0.5 μM
1 μM
2.5 μM
5 μM
7.5 μM
10 μM
17.5 μM
25 μM
35 μM
50 μM
75 μM
100 μM

Figure S3: GO$^{\bullet}$ kinetic curves of thiophenols **2-6** and phenols **1**, **7-10**.

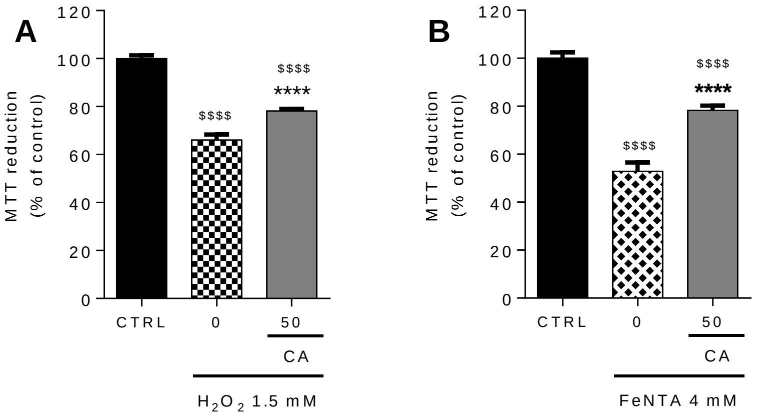

Figure S4: Protection against (**A**) H_2O_2- and (**B**) FeNTA-induced damage in differentiated SH-SY5Y cells by caffeic acid (CA). After a pre-treatment with CA (50 μM) for 24 h, cells were exposed to H_2O_2 1.5 mM and FeNTA 4 mM for additional 24 h. Cellular viability was evaluated by the MTT reduction assay. Results are expressed as the mean % of untreated controls ± SEM (n = 3). Statistical comparisons were estimated using the parametric method of one-way ANOVA, followed by the Dunnett's multiple comparisons test (**** $p < 0.001$ compared with cells treated only with FeNTA or H_2O_2; $$$$ $p < 0.0001$ compared with CTRL).

Compound **3**

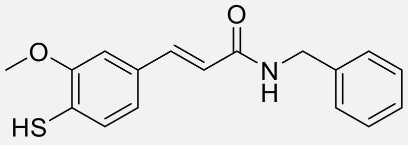

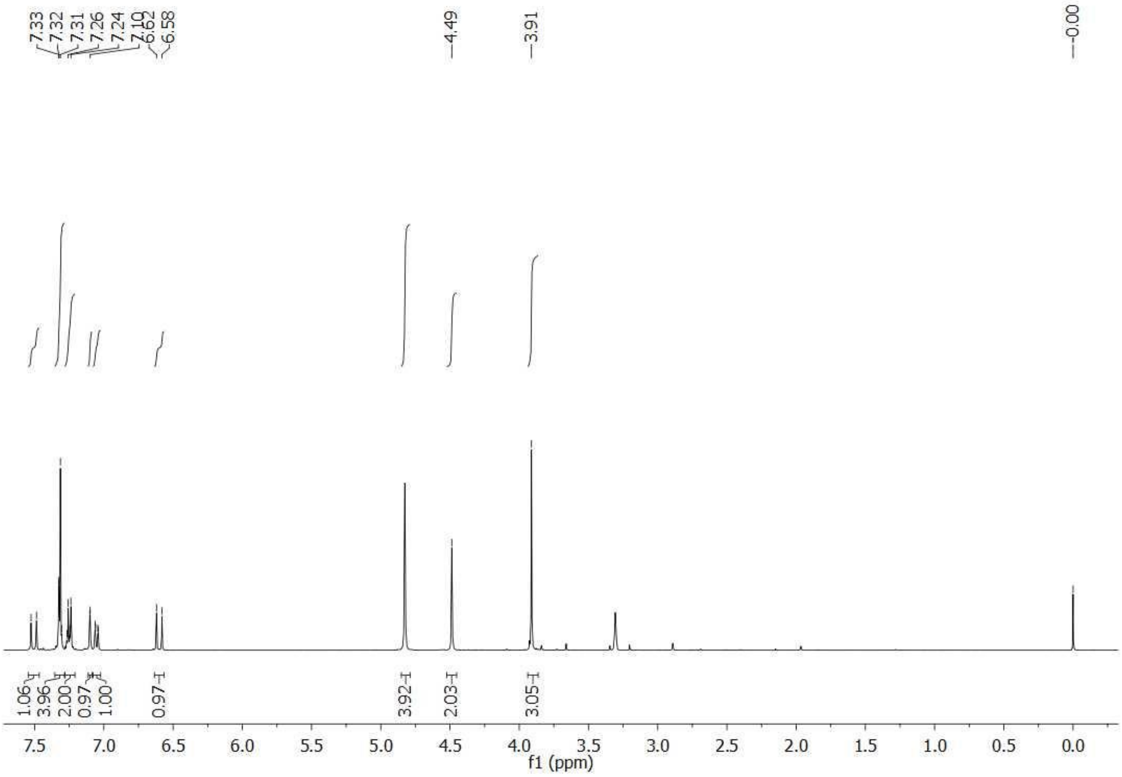

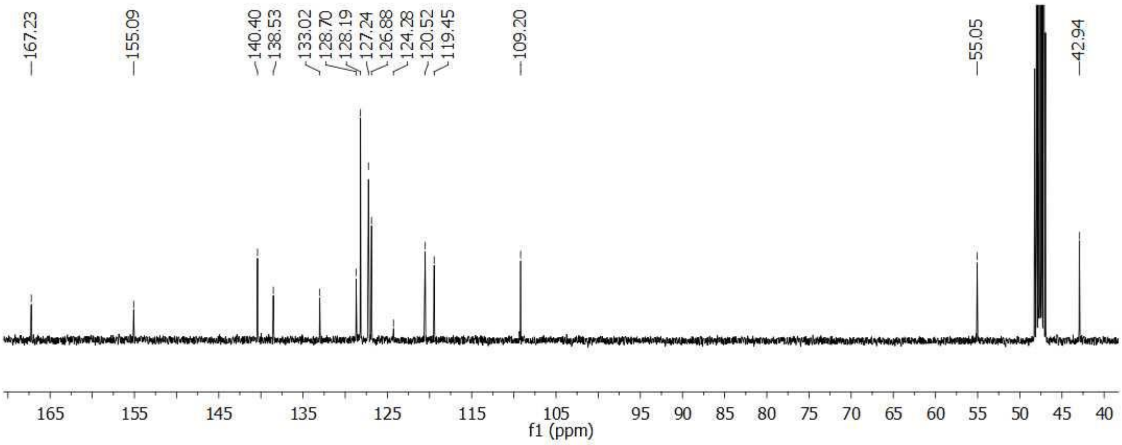

Figure S5: ^{1}H and ^{13}C NMR spectra of final compound **3** (NMR spectrum obtained in MeOD-d_4).

Compound **4**

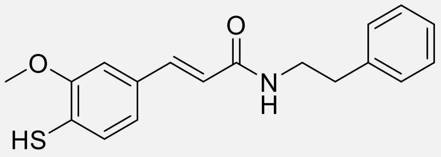

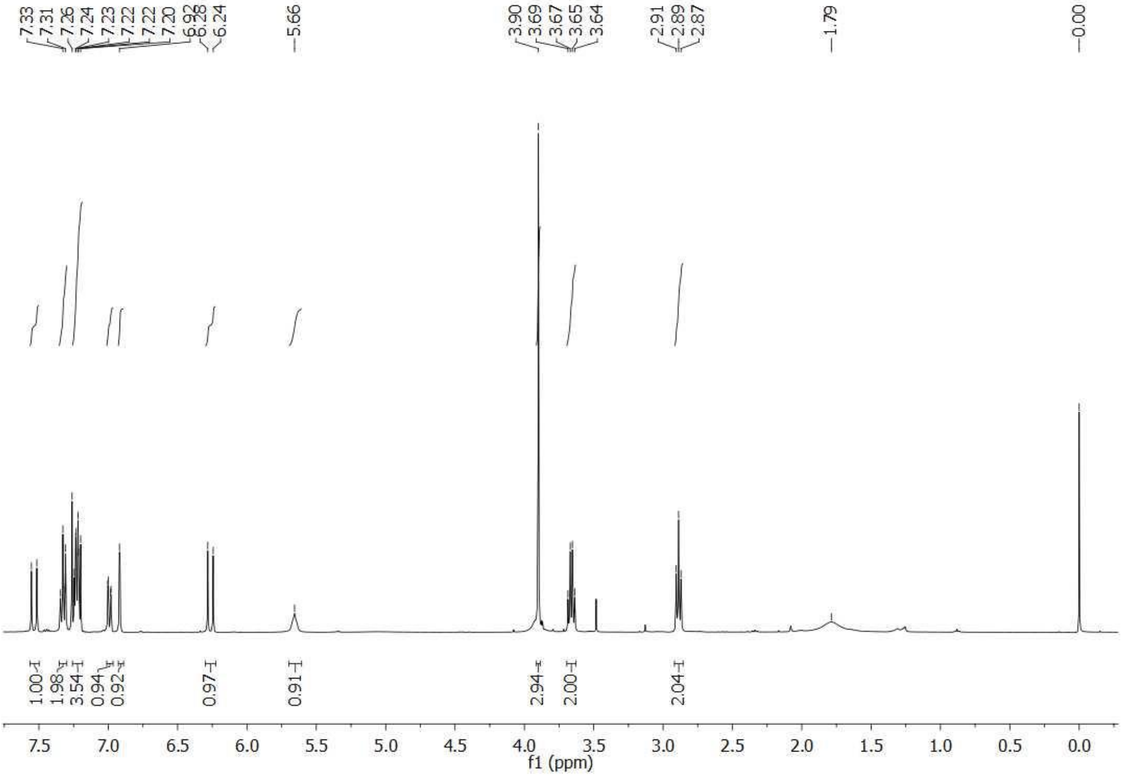

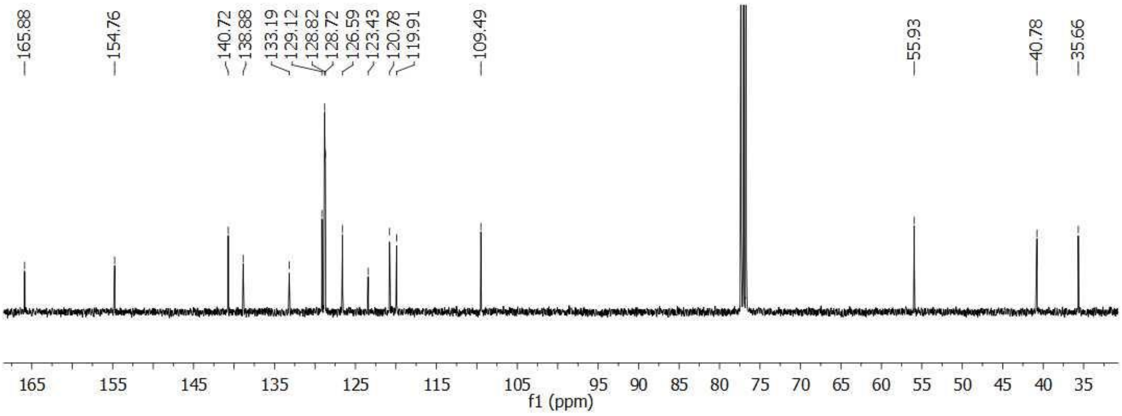

Figure S6: ^{1}H and ^{13}C NMR spectra of final compound **4** (NMR spectrum obtained in MeOD-d_4).

Compound **5**

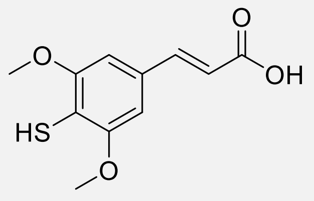

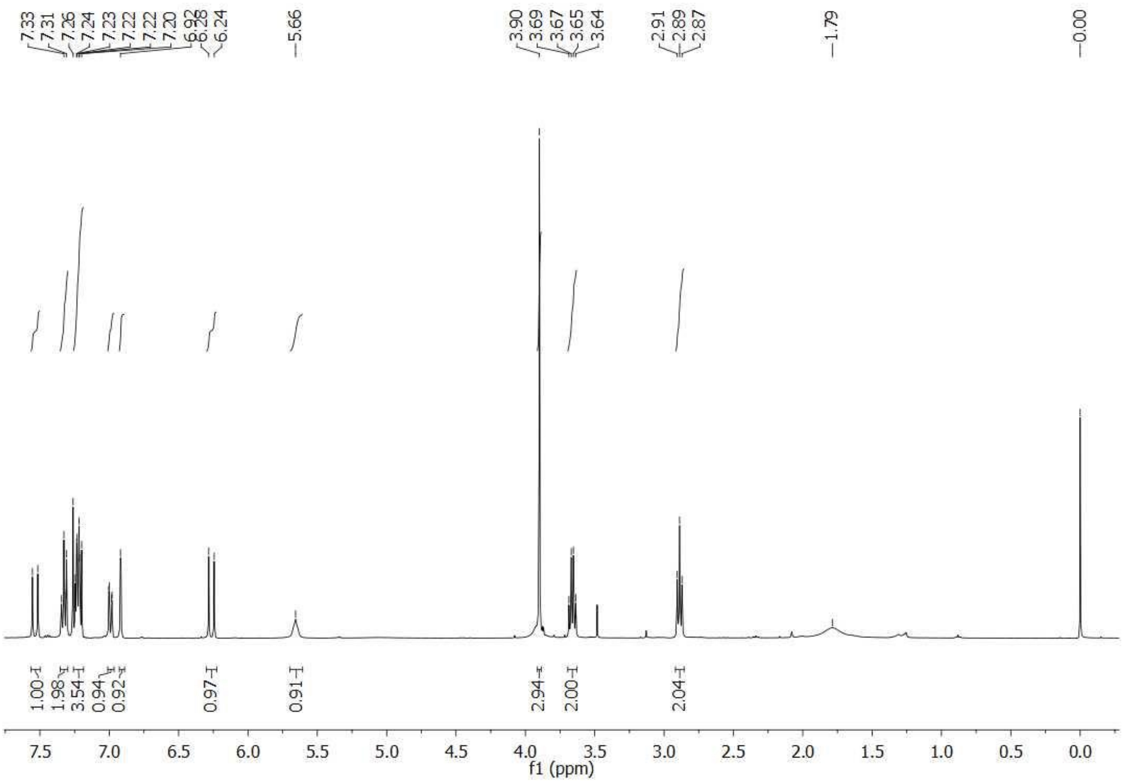

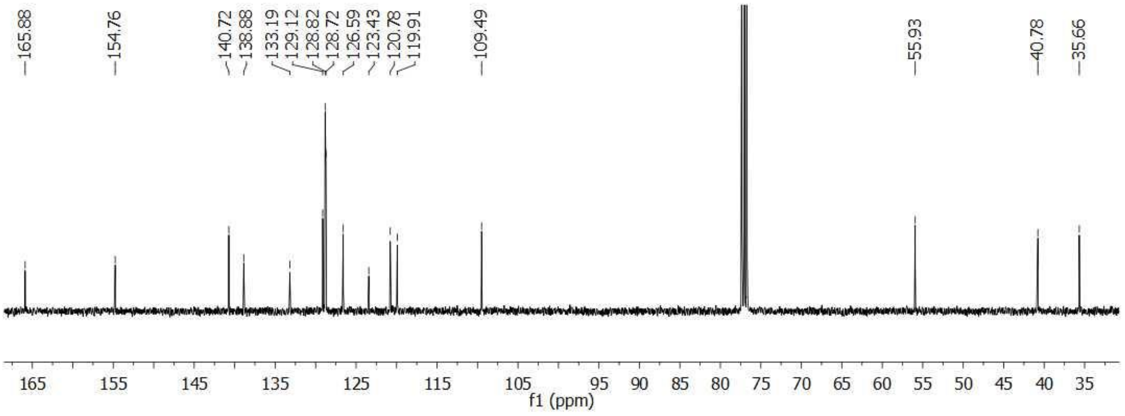

Figure S7: ^{1}H and ^{13}C NMR spectra of final compound **5** (NMR spectrum obtained in MeOD-d_4).

Compound **6**

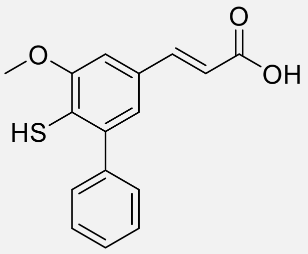

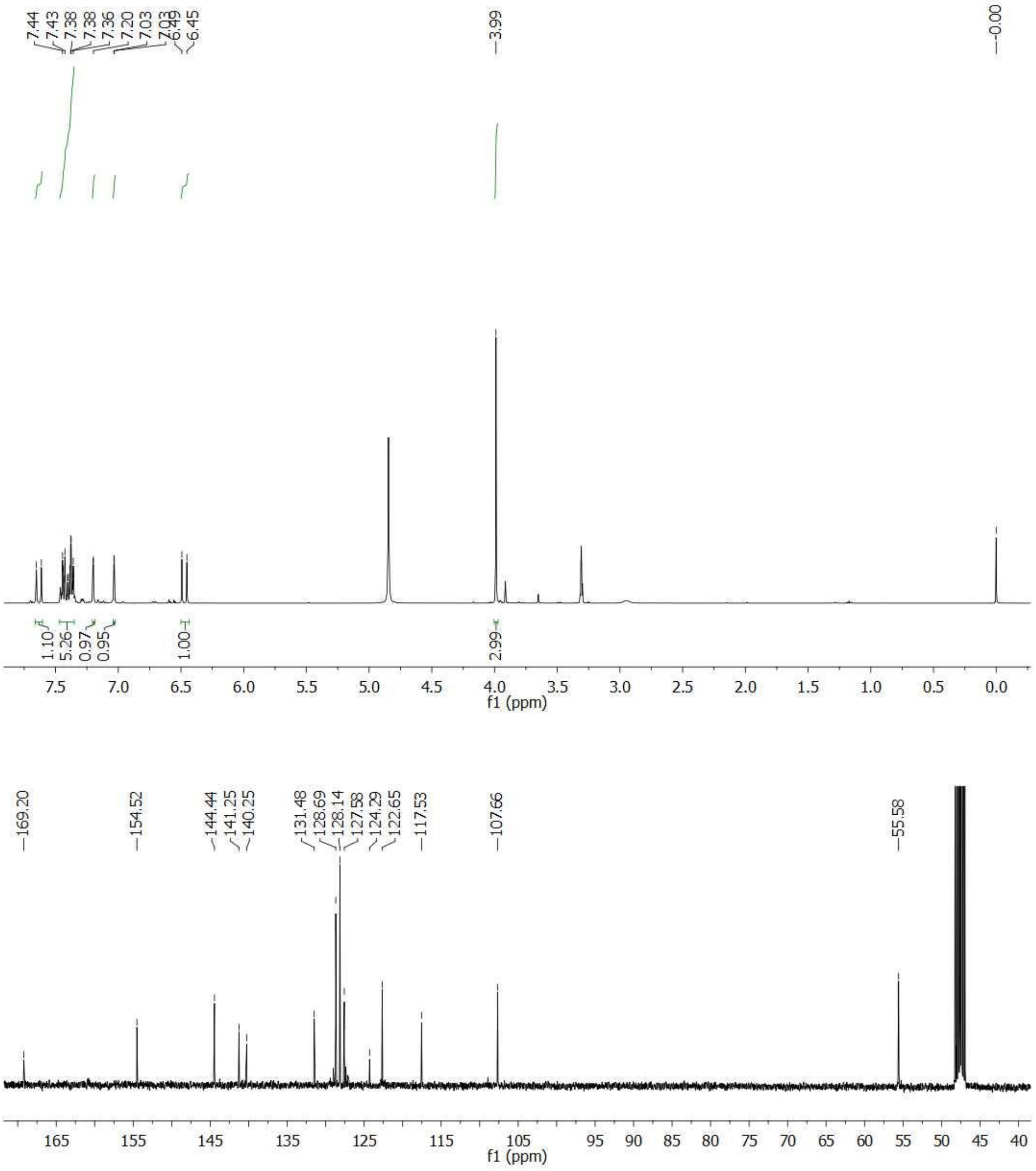

Figure S8: ^{1}H and ^{13}C NMR spectra of final compound **10** (NMR spectrum obtained in MeOD-d_4).

3. References

[1] J. Teixeira, T. Silva, S. Benfeito, A. Gaspar, E.M. Garrido, J. Garrido, F. Borges, Exploring nature profits: development of novel and potent lipophilic antioxidants based on galloyl-cinnamic hybrids, Eur. J. Med. Chem., 62 (2013) 289-296. https://doi.org/10.1016/j.ejmech.2012.12.049

[2] A. Tai, T. Sawano, H. Ito, Antioxidative properties of vanillic acid esters in multiple antioxidant assays, Biosci. Biotechnol. Biochem., 76 (2012) 314-318. https://doi.org/10.1271/bbb.110700

[3] J.Y. Feng, Z.Q. Liu, Phenolic and enolic hydroxyl groups in curcumin: which plays the major role in scavenging radicals?, J. Agric. Food Chem., 57 (2009) 11041-11046. https://doi.org/10.1021/jf902244g

[4] Y. Yang, Z.G. Song, Z.Q. Liu, Synthesis and antioxidant capacities of hydroxyl derivatives of cinnamoylphenethylamine in protecting DNA and scavenging radicals, Free Radic. Res., 45 (2011) 445-453. https://doi.org/10.3109/10715762.2010.540576

[5] B. Ou, M. Hampsch-Woodill, R.L. Prior, Development and validation of an improved oxygen radical absorbance capacity assay using fluorescein as the fluorescent probe, J. Agric. Food Chem., 49 (2001) 4619-4626. https://doi.org/10.1021/jf010586o

[6] F. Mura, T. Silva, C. Castro, F. Borges, M.C. Zuniga, J. Morales, C. Olea-Azar, New insights into the antioxidant activity of hydroxycinnamic and hydroxybenzoic systems: spectroscopic, electrochemistry, and cellular studies, Free Radic. Res., 48 (2014) 1473-1484. https://doi.org/10.3109/10715762.2014.965702

[7] J. Garrido, A. Gaspar, E.M. Garrido, R. Miri, M. Tavakkoli, S. Pourali, L. Saso, F. Borges, O. Firuzi, Alkyl esters of hydroxycinnamic acids with improved antioxidant activity and lipophilicity protect PC12 cells against oxidative stress, Biochimie, 94 (2012) 961-967. https://doi.org/10.1016/j.biochi.2011.12.015

[8] C. Fernandes, M. Pinto, C. Martins, M.J. Gomes, B. Sarmento, P.J. Oliveira, F. Remi ão, F. Borges, Development of a PEGylated-Based Platform for Efficient Delivery of Dietary Antioxidants Across the Blood-Brain Barrier, Bioconjug. Chem., 29 (2018) 1677-1689. https://doi.org/10.1021/acs.bioconjchem.8b00151

[9] L. Belyanskaya, P. Manser, P. Spohn, A. Bruinink, P. Wick, The reliability and limits of the MTT reduction assay for carbon nanotubes-cell interaction, Carbon, 45 (2007) 2643-2648. https://doi.org/10.1016/j.carbon.2007.08.010

[10] J.C. Stockert, A. Blazquez-Castro, M. Canete, R.W. Horobin, A. Villanueva, MTT assay for cell viability: Intracellular localization of the formazan product is in lipid droplets, Acta Histochem., 114 (2012) 785-796. https://doi.org/10.1016/j.acthis.2012.01.006

[11] G. Repetto, A. del Peso, J.L. Zurita, Neutral red uptake assay for the estimation of cell viability/cytotoxicity, Nat. Protoc., 3 (2008) 1125-1131. https://doi.org/10.1038/nprot.2008.75

[12] C. Fernandes, C. Martins, A. Fonseca, R. Nunes, M.J. Matos, R. Silva, J. Garrido, B. Sarmento, F. Remiao, F.J. Otero-Espinar, E. Uriarte, F. Borges, PEGylated PLGA Nanoparticles As a Smart Carrier to Increase the Cellular Uptake of a Coumarin-Based Monoamine Oxidase B Inhibitor, ACS Appl. Mater. Interfaces, 10 (2018) 39557-39569. https://doi.org/10.1021/acsami.8b17224
